# Dissecting the effect of ileal faecal diversion on the intestine using single‐cell sequencing

**DOI:** 10.1002/ctm2.1321

**Published:** 2023-07-03

**Authors:** Haitao Ma, Xiaolong Li, Yiyang Pan, Liucan Wang, Ben Han, Huichao Xie, Hong Zheng, Enlai Jiang, Jianghong Chen, Yunbo Li, Guangyan Ji, Yuan Qiu, Weidong Xiao

**Affiliations:** ^1^ Department of General Surgery Xinqiao Hospital Army Medical University Chongqing China; ^2^ Nutrition Department Xinqiao Hospital Army Medical University Chongqing China; ^3^ Department of Thoracic Surgery Xinqiao Hospital Army Medical University Chongqing China; ^4^ Department of Gastrointestinal Surgery The First Affiliated Hospital of Chongqing Medical University Chongqing China

**Keywords:** Crohn's disease, fibrosis, ileal faecal diversion, intestinal goblet cells, single‐cell RNA sequencing, TRPA1

## Abstract

**Background:**

Although ileal faecal diversion is commonly used in clinical settings, complications accompany it. Elucidating the intestinal changes caused by ileal faecal diversion will help resolve postoperative complications and elucidate the pathogenic mechanisms of associated intestinal disorders, such as Crohn's disease (CD). Therefore, our study aimed to provide new insights into the effects of ileal faecal diversion on the intestine and the potential mechanisms.

**Methods:**

Single‐cell RNA sequencing was performed on proximal functional and paired distal defunctioned intestinal mucosae from three patients with ileal faecal diversion. We also performed in vitro cellular and animal experiments, tissue staining and analysed public datasets to validate our findings.

**Results:**

We found that the epithelium in the defunctioned intestine tended to be immature, with defective mechanical and mucous barriers. However, the innate immune barrier in the defunctioned intestine was enhanced. Focusing on the changes in goblet cells, we demonstrated that mechanical stimulation promotes the differentiation and maturation of goblet cells through the TRPA1‐ERK pathway, indicating that the absence of mechanical stimulation may be the main cause of defects in the goblet cells of the defunctioned intestine. Furthermore, we found obvious fibrosis with a pro‐fibrotic microenvironment in the defunctioned intestine and identified that monocytes may be important targets for faecal diversion to alleviate CD.

**Conclusions:**

This study revealed the different transcription landscapes of various cell subsets and the potential underlying mechanisms within the defunctioned intestine, when compared to the functional intestine, based on the background of ileal faecal diversion. These findings provide novel insights for understanding the physiological and pathological roles of the faecal stream in the intestine.

## Graphical abstract

- Single‐cell RNA sequencing revealed the different transcription landscapes of the defunctioned intestine compared to the functional intestine based on the background of ileal faecal diversion.
- There is new intestinal homeostasis and enhanced fibrosis in the defunctioned intestine.
- Monocytes may be important targets for faecal diversion to alleviate Crohn's disease.

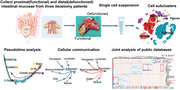

## 1 INTRODUCTION

Ileal faecal diversion is performed with an ileostomy, which forms a stoma on the abdominal wall and exudes the proximal ileum contents, typically following the surgical procedures of patients with inflammatory bowel diseases or colorectal cancer. Ileal faecal diversion has been widely performed to prevent leakage of the distal anastomosis and reduce the risk of abdominal sepsis after low pelvic anastomosis in rectal cancer surgery. Notably, ileal faecal diversion can also alleviate some intestinal diseases, such as Crohn's disease and steroid‐resistant acute graft‐versus‐host disease.^1^, ^2^ However, the incidence of complications following the reversal of ileal faecal diversion ranges from 17.3% to 21.5%.^3^, ^4^ The most frequent complications associated with the reversal of ileal faecal diversion include small bowel obstruction (4%–20%) and wound infection (5%–11%). Other complications include anastomotic leak, anastomotic stenosis and enterocutaneous fistula.^5^, ^6^, ^7^, ^8^ Diversion colitis, an inflammatory intestinal disease, is also directly related to ileal faecal diversion.^9^ Some clinical practices, such as the stimulation of the distal defunctioned intestine with faecal contents from the functional intestine, probiotics or saline before the reversal of faecal diversion, have shown promising prospects for the promotion of postoperative recovery and reduction in the incidence of complications.^10^, ^11^, ^12^ Therefore, elucidating the intestinal changes caused by ileal faecal diversion will help resolve these postoperative complications and elucidate the pathogenic mechanisms of some associated intestinal diseases.

After ileal faecal diversion, the distal defunctioned intestine is deprived of faecal stream stimulation for several months, including mechanical force, microbes and microbial metabolites. Consequently, it forms a natural contrast to the proximal functional intestine, providing a favourable window for studying the effect of faecal diversion on the intestine. Although the environment of the distal defunctioned intestine is similar to that seen in patients with parenteral nutrition, enteral nutrition from the proximal intestine can feed the distal defunctioned intestine through the mesentery. Morphological studies have revealed that the distal defunctioned intestine undergoes mucosal and muscular atrophy and luminal shrinkage, with some loss of motility and contractility.^13^, ^14^ Bulk RNA analysis also indicated reduced proliferation of the epithelium and damaged barriers in the distal defunctioned intestine.^15^, ^16^ However, the effects of ileal faecal diversion on the intestinal tract are extensive and complex, and a systematic description of microscopic alterations in the distal defunctioned intestine is lacking. Single‐cell RNA sequencing (scRNA‐seq) provides a method for the simultaneous characterization of the transcriptional state of thousands of cells. It is widely used for the analysis of various diseases and normal ecology through unbiased characterization of cells within biological tissues.^17^, ^18^, ^19^, ^20^ Therefore, we constructed a systemic and unbiased transcriptomic landscape of proximal functional and distal defunctioned intestinal mucosae. Our single‐cell study aimed to provide new insights into the effects of ileal faecal diversion on the intestine and their potential mechanisms. This has possible implications for postoperative recovery from ileal faecal diversion and the treatment of intestinal diseases.

## 2 METHODS

### 2.1 Sample collection and patient characteristics

**FIGURE 1:**
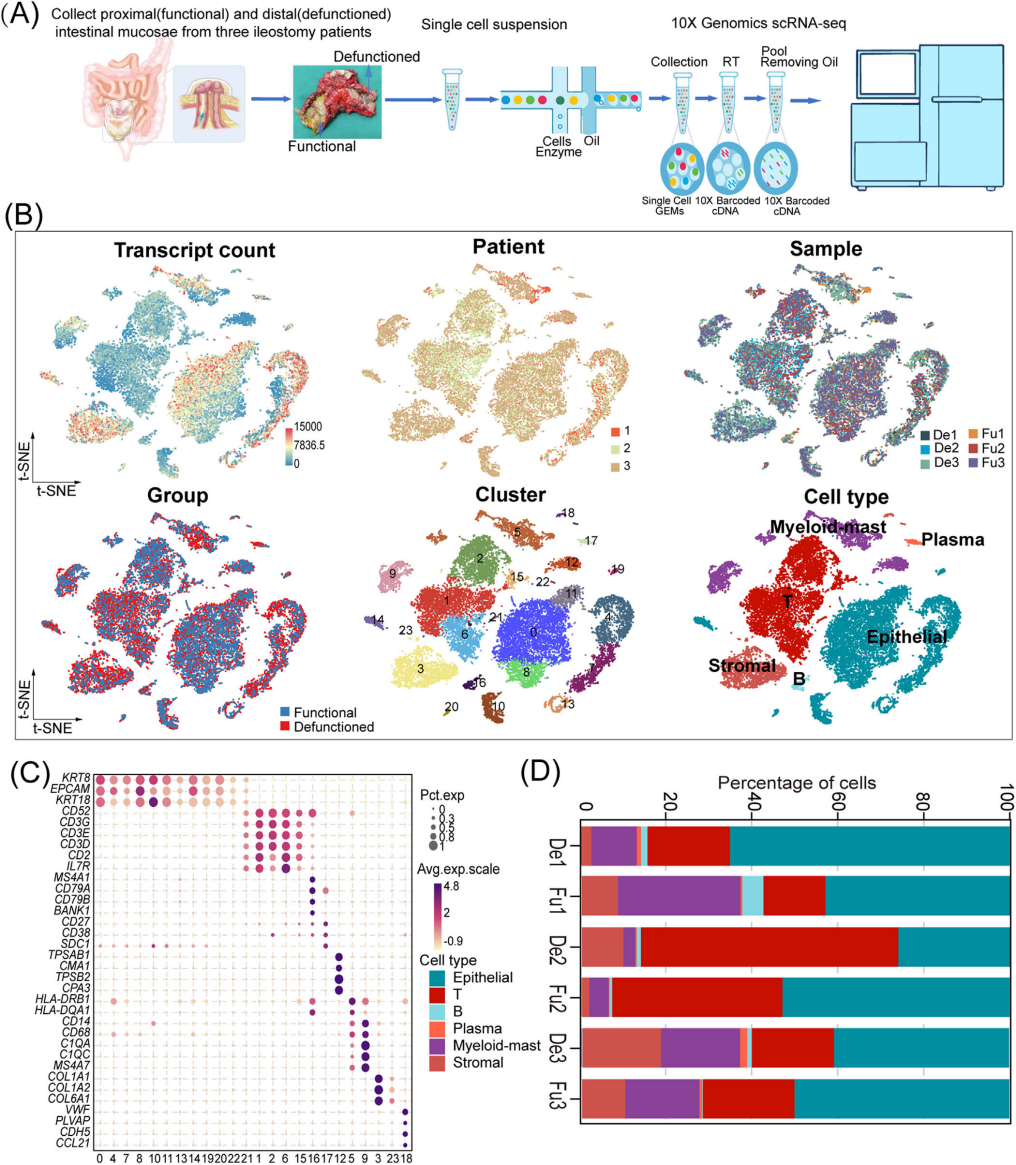
A single‐cell atlas of functional (Fu) and defunctioned (De) intestinal mucosae. (A) Workflow depicting the collection and processing of specimens of Fu and De intestinal mucosae for single‐cell RNA sequencing analysis. (B) t‐Stochastic neighbor embedding (tSNE) plots for the 21 245 high‐quality cells showing distributions of transcript counts, patients, samples, groups, cell clusters and cell types. (C) Dot plot showing the smoothed expression distribution of marker genes in six cell types. (D) Proportion of each cell type in six samples.

The proximal and distal intestines after ileostomy were defined as the functional and defunctioned intestines, respectively. During ileostomy reversal surgery, partial functional and defunctioned intestinal tissues need to be resected (Figure 1A). With the approval of the Ethics Committee of Xinqiao Hospital, Army Medical University (2020‐YD056‐01), we collected functional and defunctioned intestinal tissues for scRNA‐seq from three patients with ileal faecal diversion due to rectal cancer. The clinical characteristics of the patients, including height, weight, sex, age and basic disease, are listed in Table S1.

### 2.2 Mice

Male and female heterozygous Trpa1^+/−^ mice (C57BL/6N‐Trpa1 em1cyagen; Cyagen Biosciences, Santa Clara, CA, USA) were crossbred to generate homozygous Trpa1^−/−^ mice and wild‐type littermates. Further experiments were conducted using 8‐week‐old male mice. The terminal ileum of the mice was used for histological staining and observation. Experimental protocols involving mice were conducted according to the guidelines of the National Institutes of Health Guide for the Care and Use of Laboratory Animals and approved by the Welfare and Ethics Committee of the Army Medical University, Chongqing, China.

### 2.3 Sample preparation and scRNA‐seq

The intestinal mucosae were carefully peeled off from freshly obtained intestinal tissues, rinsed with Hank's Balanced Salt Solution (HBSS; Beyotime, Shanghai, China) and shredded into smaller pieces on ice. The shredded tissues were then incubated with RPMI (1 mg/mL collagenase IV, 2 mg/mL collagenase II, 2 mg/mL dispase and .05 mg/mL DNAse I; Invitrogen, Waltham, MA, USA) for 40 min at 37°C with gentle shaking. A 70‐mm nylon mesh filter was used to filter digested tissues, and the cell suspension was centrifuged at 350 × *g* for 5 min at 4°C. After the removal of the supernatant, we suspended the pelleted cells in red blood lysis buffer to remove red blood cells, washed them with HBSS and resuspended them in buffer (.04% bovine serum albumin [BSA] + PBS). A small amount of single‐cell suspension was added to an equal volume of .4% trypan blue staining solution (Solarbio, Beijing, China), and the cells were counted with Countess II Automated Cell Counter (Invitrogen). The concentration of live cells was adjusted to the ideal concentration (1000–2000 cells/μL). We captured single cells in Chromium Single Cell 3′ Solution, and RNA‐seq libraries were prepared using a Chromium Next GEM Single Cell 3′ Reagent Kit following the manufacturer's protocol (both 10× Genomics, Pleasanton, CA, USA). Libraries were sequenced on an Illumina NovaSeq 6000 system at Gene Denovo Biotechnology Co., Ltd. (Guangzhou, China).

### 2.4 Preprocessing of scRNA‐seq data

We used the Cell Ranger software pipeline (version 3.1.0, 10× Genomics) to process raw data from 10× Genomics. The analysis process consists of two steps: ‘cellranger mkfastq’ was applied to demultiplex raw base call files into FASTQ files, and ‘cellranger count’ was used to align FASTQ files to the reference genome, filter low‐quality data, count barcodes and unique molecular identifiers (UMIs) and generate quantitative matrices.

### 2.5 scRNA‐seq data analysis

We used Seurat (version 3.1.1)^21^ to perform scRNA‐seq data analysis. To select high‐quality cells, we used the following filtering criteria: The number of expressed genes was lower than 500 or larger than 4000; a single cell contained more than 15 000 UMIs; 25% or more of UMIs were mapped to mitochondrial genes. Those cells that met at least one of the criteria were excluded. Doublet GEMs were filtered out using the DoubletFinder tool (version 2.0.3), and batch effects were corrected using the Harmony algorithm with developer‐recommended parameters. After quality standardization, the dimensions were reduced using principal component analysis. Cell clustering analysis and gene expression marker detection were carried out using the ‘FindClusters’ (‘resolution’ = .5) and ‘FindAllMarkers’ (‘min.pct’ = .1, ‘logfc.threshold’ = 1, test.use = ‘wilcox’) functions. The ‘FindAllMarkers’ (‘min.pct’ = .1, ‘logfc.threshold’ = .1, test.use = ‘wilcox’) function was also used to perform differential analysis; t‐stochastic neighbor embedding (tSNE) was performed to visualize the clustering results of the cells.

### 2.6 Trajectory analysis

Trajectory analysis was performed using the R package Monocle2 (version 2.28.0)^22^ to explore the evolutionary trajectory of the cell subtypes. First, the monocle subject was constructed using the function ‘newCellDataSet’ (expressionFamily = negbinomial.size()). Next, a trajectory analysis was conducted on differentially expressed genes identified using Seurat. Finally, the ‘reduceDimension’ functions (method = ‘DDRTree’) were used to reduce the dimensions, and the ‘orderCells’ function was used for the construction of pseudotime trajectory and alignment of cells within the pseudotime time.

### 2.7 Transcription factor analysis

The SCENIC R package^23^ was used to carry out transcription factor network inference. Briefly, the input matrix was a log‐normalized expression matrix generated using Seurat, and three steps were executed to implement the pipeline. First, we used GENIE3 (version 1.4.3) to identify a gene co‐expression module. According to a regulatory motif near the transcription start site, we then used RcisTarget (version 1.2.1) to predict the significantly enriched transcription factor (TF)‐binding motifs in the module and select genes highly associated with the motifs in the module as target genes. The TFs and target genes retained in the module were constructed as a regulon. Third, we utilized AUCell (version 1.4.1) to evaluate the activity of each regulon for each cell using the AUC values. These programmes were all run with default parameters. We also obtained all TFs from the Animal Transcription Factor Database (http://bioinfo.life.hust.edu.cn/AnimalTFDB/#!/download) and used the ‘FindAllMarkers’ (test.use = ‘wilcox’) function in Seurat to select highly expressed TFs in different intestinal epithelial cells.

### 2.8 Cell‐to‐cell communication of scRNA‐seq data

We performed ligand–receptor analysis using the CellPhoneDB software.^24^ The expression matrix and cellular annotations generated by Seurat were used as input files. Receptors and ligands expressed by more than 10% of the cell populations were included in the analysis. To characterize biological interactions, we separately performed pairwise comparisons between all cell subpopulations of the functional and defunctioned intestines in our data. We also identified significantly enriched ligand–receptor pairs between cell subpopulations and compared them between the functional and defunctioned intestines according to the mean expression of ligand–receptor pairs and *p*‐values. In addition, a network map of intercellular interactions was constructed to illustrate the regulatory relationships between cells.

### 2.9 Functional enrichment analysis

We performed gene set enrichment analysis (GSEA), gene ontology (GO) and Kyoto Encyclopedia of Genes and Genomes (KEGG) enrichment analyses using the R package ClusterProfiler (version 3.10.1). The R package GSVA (version 1.30.0) was used to perform gene set variation analysis (GSVA), and gene sets were collected from the Molecular Signatures Database (MSigDB; www.gsea‐msigdb.org).

### 2.10 Collection of CD‐related genes

We collected 1505 CD‐related genes from DisGeNET^25^ (https://www.disgenet.org/home/) and Gene2Function^26^ (https://www.gene2function.org/search/) (Table S10). We also collected CD‐related genes from two public microarray datasets (GSE186582 and GSE95095) that included data from normal and CD tissues. We eliminated batch effects between the microarray datasets using the R package sva (version 3.30.1). By performing differential analysis using limma R package (version 3.22.7), we obtained 5805 CD‐related differential genes (Table S10). Then, 579 genes significantly related to CD were obtained from the intersection of the two gene sets.

### 2.11 Immunofluorescence, immunohistochemistry and in situ hybridization

The sections were conventionally deparaffinised, rehydrated and subjected to antigen retrieval. Subsequently, we blocked sections with 3% BSA at room temperature for 30 min. Next, we incubated the sections with primary antibodies overnight in blocking buffer at 4°C. The primary antibodies used are as follows: anti‐occludin (1:100; Proteintech, IL, USA), anti‐DEFA6 (HD6, 1:5000; Atlas, Bromma, Sweden), anti‐DEFA5 (HD5, 1:50; Abcam, Cambridge, UK), anti‐ZO1 (1:200; Abcam), anti‐LYZ (1:100; Invitrogen), anti‐Chromogranin A (CHGA, 1:200; Abcam), anti‐OLFM4 (1:100; CST, Danvers, MA, USA), anti‐pERK (1:100; CST), anti‐MUC2 (1:500; Abcam), anti‐TRPA1 (1:100; Proteintech), anti‐TFF1 (1:200; Abcam), anti‐SPDEF (1:25; Santa Cruz Biotechnology, Dallas, TX, USA), anti‐PTGS1 (1:250; Abcam) and anti‐THY1 (1:150; Abcam). The following day, secondary antibodies labelled with fluorescein (1:300; Life Technologies, CA, USA) for immunofluorescence or biotin (1:100; Zhongshan, Beijing, China) for immunohistochemistry (IHC) were added at room temperature for 2 h. Nuclei were mounted with 4′,6‐diamidino‐2‐phenylindole (DAPI, 1:1000; Beyotime) for immunofluorescence, and reactions were visualized by H_2_O_2_/DAB solution for IHC. The human LGR5 (NM_003667.2) in situ hybridization kit was purchased from Boster Bio‐engineering Co (Wuhan, China), and the procedure was performed according to the manufacturer's protocol. We captured all histological images under a light microscope (Leica, Wetzlar, Germany). In order to quantify cells, we evaluated at least 10 villi or crypts per sample from five fields of view. The mean ratios of the THY1 area to mucosa area were obtained by taking 10 measurements within five fields of view per sample using Image Pro Plus 6.0 software. The mean ratios of integrated optical density (IOD) to positive area (IOD/pixel) were used to quantify the relative expression of proteins using Image Pro Plus 6.0. The value (IOD/pixel) was obtained by taking 10 measurements within five fields of view per sample.

### 2.12 Alcian blue/periodic acid‐Schiff staining and Masson's trichrome staining

Alcian blue/periodic acid‐Schiff (AB–PAS) stain and Masson's trichrome stain kits (Solarbio) were used on 5 mm, formalin‐fixed, paraffin‐embedded sections following the manufacturer's instructions. Image Pro Plus 6.0 was used to measure the thickness of the mucosal muscularis, muscularis propria and mucus layer. A total of 10 measurements were taken within three fields of view per sample to obtain the average thickness. We created a simple fibrosis scoring criterion based on a previous study^27^: 0, no increased collagen deposition; 1, increased collagen deposition in the mucosa; 2, increased collagen deposition in the mucosa and muscularis mucosa; and 3, increased collagen deposition in the mucosa, muscularis mucosa and submucosa. The average fibrosis score was obtained by performing five evaluations within five fields of view per sample.

### 2.13 Transmission electron microscopy

According to a previously described procedure, samples were prepared for transmission electron microscopy (TEM).^28^ Tissues were standardly fixed, dehydrated, permeabilized and embedded. Uranyl acetate and lead citrate were used to stain the tissues cut at 60‐nm intervals. We observed tissues using a TEM (JEM 1200EX; JEOL, Tokyo, Japan). Mucin granules were quantified by evaluating more than three different fields of view per sample.

### 2.14 Cell culture

Dulbecco's modified Eagle's medium (Gibco, YN, USA), containing 1% penicillin/streptomycin (Gibco) and 10% FBS (Invitrogen), was used to culture the human CRC‐derived LS174T cell line (ATCC, Manassas, VI, USA) at 37°C in a 5% CO_2_ incubator. After fusion, the cells were used for further experiments. Cells were placed on a rocking board with fixed parameters to adapt to mechanical stimulation. Cells were treated with ASP7663 (5 μM; MCE, NJ, USA) and HC‐030031 (5 μM; MCE) to activate or inhibit TRPA1. PD98059 (10 μM; MCE) was used to block ERK signalling activity.

### 2.15 Transfection assays

TRPA1 knockdown was achieved by infecting LS174T cells with lentivirus‐packaged shRNA (Shanghai Genechem Co., Ltd., China). Lentiviruses containing negative control shRNA were used in the control group. Purified GFP‐positive cells were passaged at least twice in culture medium containing .5% puromycin (Genechem) until they reached 99% confluency.

### 2.16 Western blot

A RIPA lysis buffer, including inhibitors of phosphatase and protease (Beyotime), was used to extract total proteins. We then transferred the proteins to polyvinylidene difluoride (PVDF) membranes (Millipore, Burlington, MA, USA) following sodium dodecyl sulphate–polyacrylamide gel electrophoresis. Next, we blocked the PVDF membranes with 5% non‐fat dry milk for 1 h and washed them three times with tris‐buffered saline containing .05% Tween‐20 (TBST). We then incubated the PVDF membranes with primary antibodies overnight at 4°C. After three washes with TBST, we incubated the PVDF membranes with the corresponding secondary antibody conjugated to HRP (Abcam) for 1 h at room temperature. Rabbit anti‐GAPDH antibody (Proteintech) was used as an internal control. Enhanced chemiluminescence kit (Beyotime) was used to detect proteins, and the ImageJ software was used to analyse their intensity. The primary antibodies used in this study are as follows: anti‐MUC2 (1:1000; Abcam), anti‐SPDEF (1:500; Santa Cruz Biotechnology), anti‐ERK (1:2000; CST), anti‐pERK (1:2000; CST), anti‐TRPA1 (1:500; Proteintech) and anti‐ATOH1 (1:1000; Invitrogen).

### 2.17 Isolation of epithelial cells and fibroblasts

The epithelial cells and fibroblasts derived from the functional and defunctioned intestines were isolated as previously described.^29^, ^30^ The functional and defunctioned intestinal tissues were obtained from patients with ileal faecal diversion due to rectal cancer. We cut the intestinal tissues into small pieces and incubated them with HBSS‐EDTA at 37°C for 30 min. Epithelial cells were obtained by centrifuging the suspension at 400 × *g* for 5 min at 4°C. The remaining intestinal tissues were used for fibroblast isolation and digested with an enzyme mix containing hyaluronidase (2 mg/mL), DNAse (1 μL/mL) and collagenase I (1 mg/mL) (Invitrogen) until the samples were fully dissolved at 37°C. Then we used a 70‐μm nylon strainer to filter the digested tissues. The cells were cultured in a FGMTM‐2 Fibroblast Growth Medium‐2 (Lonza, Basel, Switzerland) containing 1% penicillin/streptomycin.

### 2.18 Real‐time quantitative PCR

We isolated total RNA from the cells using TRIzol reagent (TaKaRa Biology Inc., Kusatsu, Shiga, Japan), and cDNA was acquired using MIX reverse transcription primer (TaKaRa Biology Inc.). Real‐time quantitative PCR was performed on a LightCycler 480 using SYBR Green (Roche Diagnostics, Basel, Switzerland). We evaluated the relative expression of genes using the 2^−ΔΔ^ *^Ct^* method [ΔΔ*Ct* = (*Ct* target − *Ct* reference) sample − (*Ct* target − *Ct* reference) control]. The primer sequences we used in this study are listed in Table S9.

### 2.19 Statistical analysis

R software (version 4.1) and GraphPad Prism (version 9.0) were used to perform statistical analyses. Histological measurements, cell counts, PCR gene expression values and protein levels quantified using IHC for human samples were compared using the paired Wilcoxon rank‐sum test. Cell counts in mice were compared using unpaired Wilcoxon rank‐sum test. The Wilcoxon rank‐sum test in the Seurat package was used to analyse differential gene expression in different cell subpopulations. Protein expression quantified by western blot was compared using unpaired *t*‐test. Statistical significance was set at *p* < .05.

## 3 RESULTS

### 3.1 High‐resolution cell‐type mapping of proximal (functional) and distal (defunctioned) intestinal mucosae

Intestinal mucosae were isolated from paired functional and defunctioned intestines obtained from surgically resected terminal ileal tissues of three patients with ileal faecal diversion performed for rectal cancer (Table S1). We profiled single‐cell transcriptomes of these intestinal mucosae paired with a 10× Genomics system (Figure 1A). After quality filtering (see Section 2), we obtained transcriptome profiles of 21 245 cells for downstream analyses (Table S2, De1:1427, Fu1:1802, De2:3661, Fu2:4541, De3:5996 and Fu3:3818). After dimensionality reduction and unsupervized clustering, cells selected for downstream analyses were clustered into 24 clusters based on the tSNE plots (Figure 1B). In accordance with previously reported cell markers,^17^, ^18^ these clusters were divided into six cell types: epithelial (*KTR8*, *EPCAM* and *KRT18*), T (*CD3D*, *CD3G* and *CD3E*), B (*MS4A1*, *CD79B*, *CD79A* and *BANK1*), plasma (*CD27*, *CD38* and *SDC1*), myeloid‐mast (*TPSAB1*, *CMA1*, *TPSB2*, *CD14*, *CD68*, *C1QB*, *C1QA* and *MS4A7*) and stromal (*COL1A1*, *COL1A2*, *COL6A1*, *VWF*, *PLVAP*, *CDH5* and *CCL21*) (Figure 1B,C, Table S3) cells.

Based on previously reported cell markers and other intestinal scRNA‐seq results (Table S4),^17^, ^18^, ^20^, ^31^ T cells were subdivided into nine cell types: Th17, CD8 T, γδ cytotoxic T, γδ T, MT‐hi T,^17^ Treg/follicular helper T, ILC3s, cycling T^18^ and REG1A/1B cells^32^ (Figure S1A–C, Table S5). Myeloid‐mast cells were subdivided into five cell types: monocytes, macrophages, *LYVE1* macrophages,^31^ DC2s and DC1/pDCs (Figure S1D–F, Table S6). Stromal cells were subdivided into six cell types: fibroblasts1, fibroblasts2, glial cells, smooth muscle cells, blood endothelial cells and lymphatic endothelial cells (Figure S1G–I, Table S7). The subdivision of the epithelial cells is described in the next section.

### 3.2 Ileal faecal diversion affects epithelial maturation and barrier integrity in the defunctioned intestine

**FIGURE 2:**
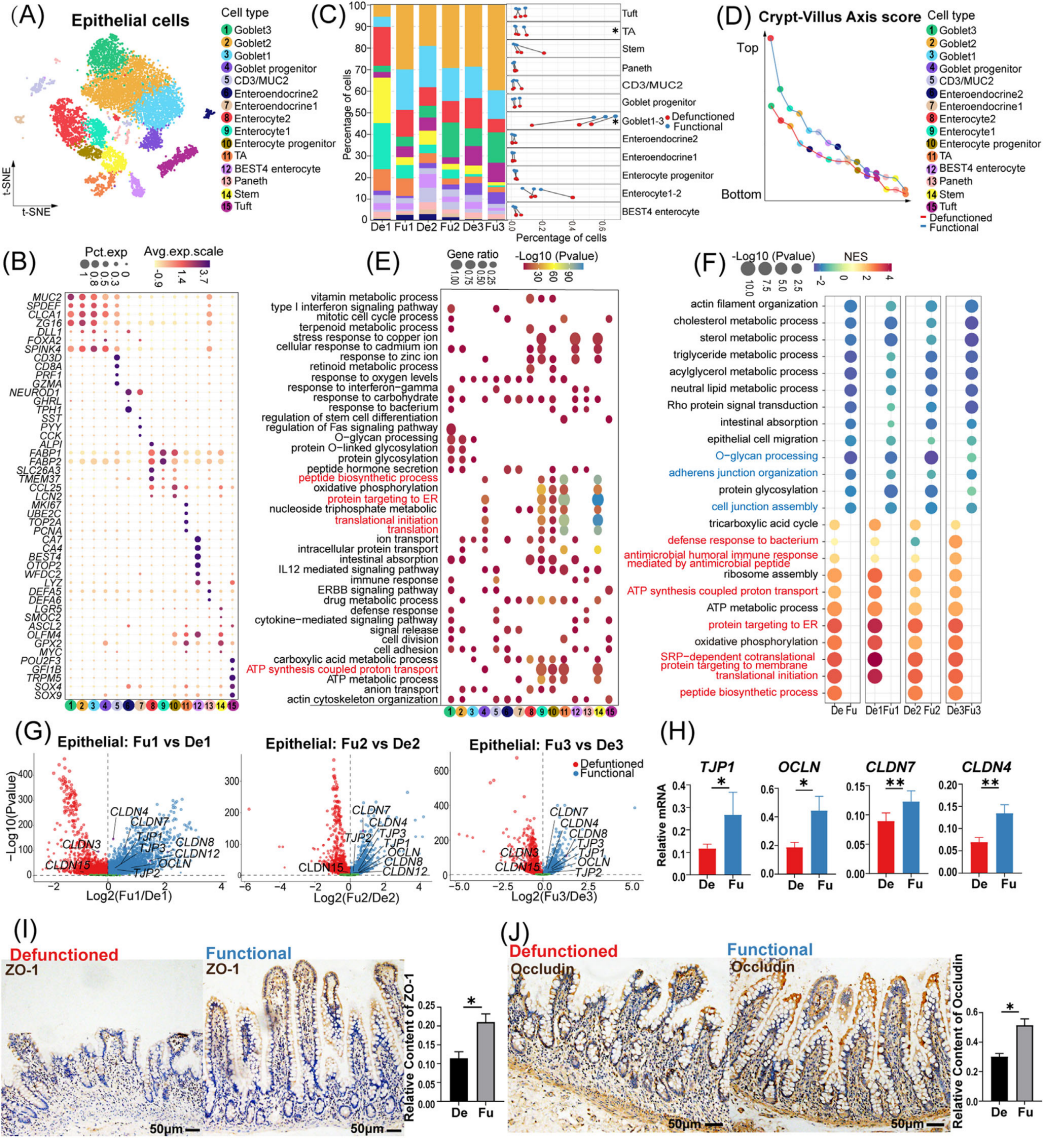
Classification and functional (Fu) annotation of epithelial cells, differences in epithelial cells between the Fu and defunctioned (De) intestines. (A) t‐Stochastic neighbor embedding (tSNE) plot showing 15 subsets of epithelial cells. (B) Dot plot showing the expression of significant marker genes in 15 cell types. (C) Proportion of each epithelial cell subset in the Fu and De intestines (*p < .05, paired *t*‐test). (D) Crypt–villus axis curve of the Fu and De intestines. Crypt–villus axis scores indicate the distance from the bottom of the crypt. (E) Gene ontology enrichment of marker genes (log2FC > 1, *p* < .05, Wilcoxon rank‐sum test) in each cell type. (F) Gene set enrichment analysis showing enriched biological pathways in epithelial cells of the Fu and De intestines (*p* < .05). (G) Volcano plots showing significant differential expression of genes related to tight junction between epithelial cells of the Fu and De intestines (*p* < .05, Wilcoxon rank‐sum test). (H) Relative mRNA expression of *TJP1*, *OCLN*, *CLDN4* and *CLDN7* in epithelial cells of the Fu and De intestines was confirmed using quantitative PCR (*p < .05, **p < .01, *n* = 7). (I and J) Immunohistochemistry staining of ZO‐1 and occludin in the De and Fu intestines (*p < .05, *n* = 5, scale bars = 50 μm). (H–J) Paired Wilcoxon rank‐sum test. All values are presented as mean ± SEM of each group. NES, normalized enrichment score.

Intestinal epithelial cells were subdivided into 15 clusters: goblet cells1–3, goblet progenitors, CD3/MUC2 cells, enteroendocrine cells1–2, enterocytes1–2, BEST4 enterocytes, enterocyte progenitors, stem cells, tuft cells, transit‐amplifying (TA) cells and Paneth cells (Figure 2A,B, Table S8). Among enterocytes, three clusters showed distinct developmental trajectories according to trajectory analysis (Figure S2A). GSVA showed that lipid metabolic pathways (such as VLDL assembly) were more enriched in enterocytes2; enterocytes1 had more enriched bile acid metabolic pathway (FXR pathway); BEST4 enterocytes had strong heterogeneity and were enriched for pathways associated with ion transport (such as zinc homeostasis and response to metal ions) (Figure S2C). The development of BEST4 enterocytes was more dependent on the HES pathway, with a high expression of *HES4*, *HES5* and *NOTCH2* (Figure S2B,C). We identified three enteroendocrine clusters based on dimensionality reduction and hormone expression: enterochromaffin, I/L/N and D cells (Figure S2D).^20^ Further quantitative PCR analysis showed decreased *NTS* expression in the epithelium of the defunctioned intestine (Figure S2E). According to our data, the proportion of goblet cells was reduced in the defunctioned intestine (Figure 2C).

Intestinal villus atrophy due to faecal diversion seems to be variable (Figure S2F), which may be related to individual differences among patients and the duration of faecal diversion. The cause of intestinal mucosal atrophy still remains unknown. Therefore, we calculated and plotted crypt–villus axis^33^ curves of the epithelium, which was lower in the defunctioned intestine than in the functional intestine (Figure 2D), suggesting that the epithelium in the defunctioned intestine may be younger. GSEA also showed that significantly enriched gene sets (such as protein targeting to ER and translational initiation) in the epithelium of the defunctioned intestine overlapped with gene sets characteristic of stem and progenitor cells (Figure 2E,F). Consistent with this, barrier, as one of the main functions of the intestinal epithelium, the gene sets associated with it (e.g. cell junction assembly and *O*‐glycan processing) were significantly down‐regulated in the defunctioned intestine (Figure 2F). Differential analysis showed that although the expression of *CLDN3* and *CLDN15* was increased, *TJP1*, *TJP2, TJP3, OCLN*, *CLDN4*, *CLDN7* and *CLDN8* expression in the epithelium of the defunctioned intestine was significantly reduced (Figure 2G). The reduction in *TJP1* (ZO‐1), *OCLN* (occludin), *CLDN4* and *CLDN7* was further confirmed using quantitative PCR or IHC staining (Figure 2H–J). The tSNE plots showed that most intestinal epithelial cells of the terminal ileum expressed *CLDN3*, *CLDN4* and *CLDN7* (Figure S2G). *CLDN8* was mainly expressed in goblet cells, and *CLDN15* was mainly expressed in enterocytes (Figure S2G). The defunctioned intestine also had a defective mucus barrier with a thin mucus layer, as confirmed by AB–PAS staining (Figure S2H). In addition, metabolism‐ and absorption‐related gene sets in the epithelium of the defunctioned intestine were significantly down‐regulated (Figure 2F).

**FIGURE 3:**
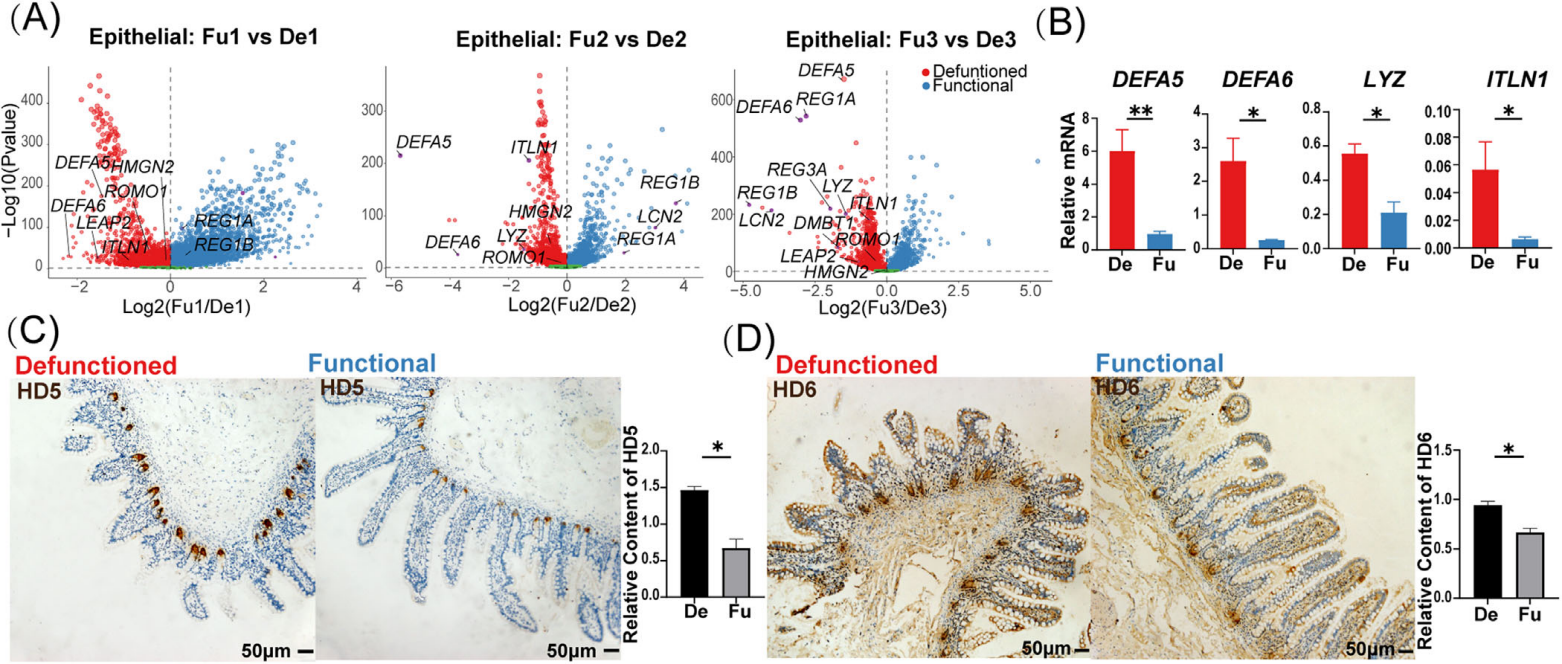
Differential expression of genes related to antimicrobial peptides in epithelial cells between the functional (Fu) and defunctioned (De) intestines. (A) Volcano plots showing significant differential expression of genes (*p* < .05, Wilcoxon rank‐sum test). (B) Relative mRNA expression of *DEFA5*, *DEFA6*, *LYZ* and *ITLN1* in epithelial cells of the Fu and De intestines was confirmed by quantitative PCR (*p < .05, **p < .01, *n* = 7). (C and D) Immunohistochemistry staining of HD5 and HD6 in the De and Fu intestines (*p < .05, *n* = 5, scale bars = 50 μm). (B–D) Paired Wilcoxon rank‐sum test. All values are presented as mean ± SEM of each group.

Unlike the mechanical and mucus barriers, which were weakened, the innate immune barrier of the defunctioned intestine was enhanced. First, we found that antibacterial‐related gene sets (such as the antimicrobial humoral immune response) were enriched in the epithelium of the defunctioned intestine (Figure 2F). Based on the gene set for antimicrobial peptides collected from MSigDB,^34^ differential analysis revealed significantly increased expression of antimicrobial peptide genes such as *DEFA5* and *DEFA6* in the epithelium of the defunctioned intestine (Figure 3A). The expression of *DEFA5* (HD5), *DEFA6* (HD6), *LYZ* and *ITLN1* was confirmed by IHC staining or quantitative PCR (Figure 3B–D). Recent studies have shown that Sh2d6‐expressing tuft‐2 cells in the intestine of mice exert antimicrobial immunity.^35^ In our data, tuft cells highly expressed *SH2D6* and the antimicrobial peptide gene *DEFB1* (Figure S2I). However, there was no significant difference in the number and *DEFB1* expression level between tuft cells of the functional and defunctioned intestines (Figure S2J,k). Enhanced innate immunity may compensate for defects in mechanical and mucous barriers, which form a new homeostasis in the defunctioned intestine.

### 3.3 Ileal faecal diversion impairs the differentiation and maturation of goblet cells in the defunctioned intestine

**FIGURE 4:**
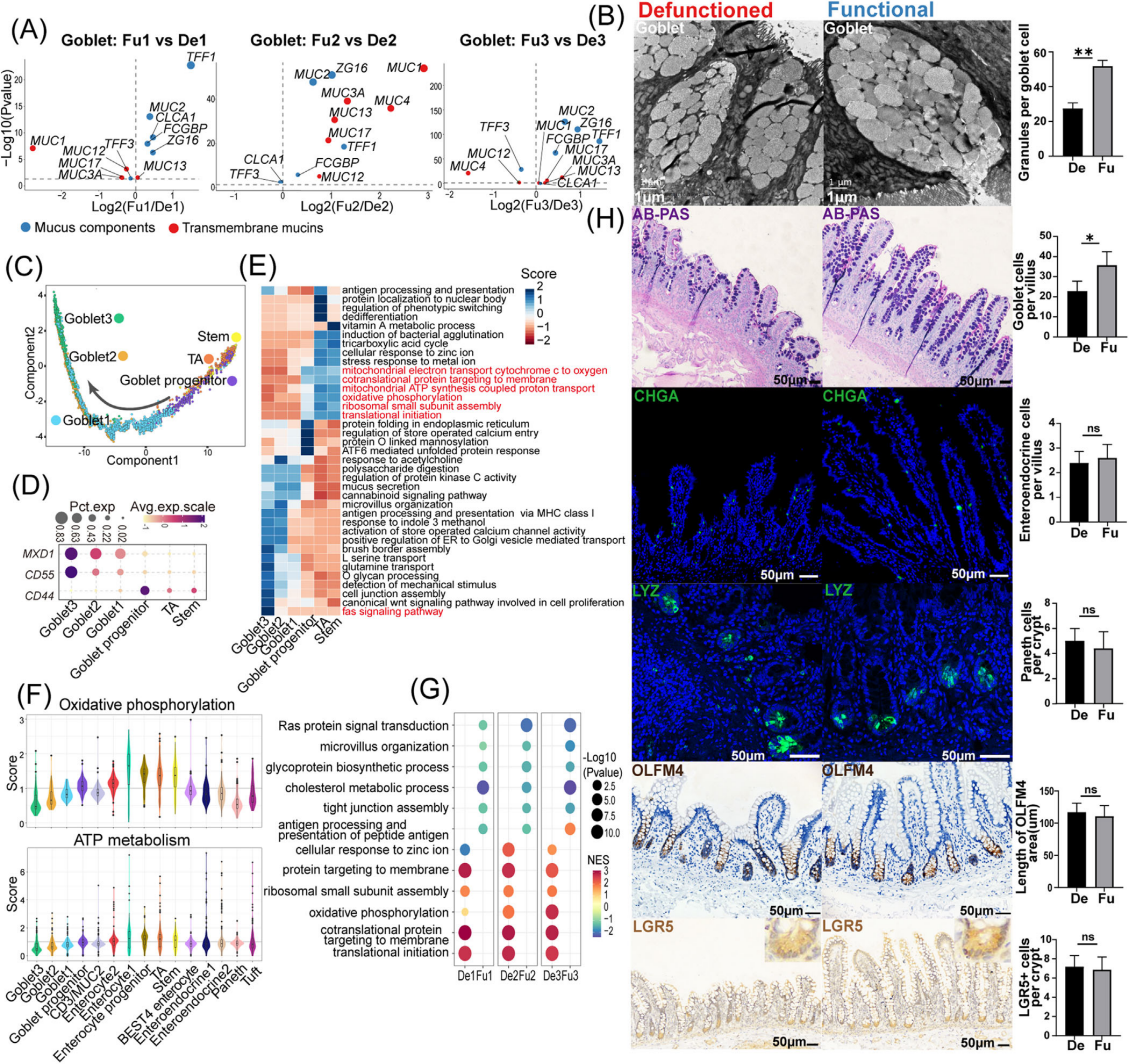
Changes in goblet cells of the defunctioned (De) intestine mediated by ileal faecal diversion. (A) Volcano plots showing significant differential expression of genes related to mucus components and transmembrane mucins in goblet cells between the functional (Fu) and De intestines (*p* < .05, Wilcoxon rank‐sum test). (B) Transmission electron microscope images showing mucin granules in goblet cells of the Fu and De intestines (**p < .01, *n* = 7, scale bars = 1 μm). (C) Monocle 2 pseudotime analysis showing the developmental trajectory of goblet cells. (D) Dot plot showing the expression of *CD44*, *CD55* and *MXD1* in goblet, transit‐amplifying (TA) and stem cells. (E) Differences in pathway activities scored per cell using gene set variation analysis in goblet, TA and stem cells. Pathway scores were normalized. (F) Violin plots showing the oxidative phosphorylation and ATP metabolism scores of each subset of epithelial cells. (G) Gene set enrichment analysis showing enriched biological pathways in goblet cells of the Fu and De intestines (*p* < .05). (H) Alcian blue/periodic acid‐Schiff (AB–PAS) staining and immunostaining of CHGA, LYZ, OLFM4 and LGR5 in the Fu and De intestines (*p < .05, ns = not significant, *n* = 5, scale bars = 50 μm). (B and H) Paired Wilcoxon rank‐sum test. All values are presented as mean ± SEM of each group. NES, normalized enrichment score.

Based on the defective mucus barrier of the defunctioned intestine, we investigated the detailed changes in goblet cells of the defunctioned intestine. Differential analysis revealed that the expression of genes related to mucus components (e.g. *MUC2*, *ZG16* and *TFF1*) and transmembrane mucins (*MUC13*) was significantly reduced in goblet cells of the defunctioned intestine (Figure 4A). The expression of MUC2 and TFF1 was confirmed by IHC staining (Figure S3A,B). Highly consistent with this, TEM images also displayed significantly reduced mucin granules in goblet cells of the defunctioned intestine (Figure 4B). Intriguingly, the expression of *TFF3* ^36^ that can promote epithelial restitution was increased in goblet cells of the defunctioned intestine, which may be a compensatory response to the defective barrier (Figure 4A). Next, trajectory analysis suggested that the order of differentiation and maturation of goblet cells was as follows: stem cells, TA cells, goblet progenitors, goblet cells1, goblet cells2 and goblet cells3 (Figure 4C). Among them, goblet progenitors exhibited high expression levels of *CD44*, which was found to be significantly expressed in intestinal stem cells and is a major direct target of Wnt signalling^37^ (Figure 4D). Goblet cells3 were the most differentiated goblet cells with a high expression of *MXD1*, a high crypt–villus axis score and enriched Fas signalling pathway (Figures 2D and 4D,E), which was similar to the inter‐crypt goblet cells found by Nyström et al.^38^ GSVA also displayed changes in the activity of goblet cells during differentiation and maturation, such as enhanced antigen presentation and decreased mitochondrial activity (Figure 4E, Figure S3C,D). Recent studies have indicated that mitochondrial activity plays an essential role in the proliferation and differentiation of intestinal stem cells.^39^, ^40^ Therefore, we calculated the scores of oxidative phosphorylation and ATP metabolism in epithelial cells using the algorithm developed by Xiao et al.^41^ The results showed that stem cells, TA cells and enterocytes had higher scores for oxidative phosphorylation and ATP metabolism. By contrast, secretory cells had the opposite effect, and the scores continued to decrease with the maturation of goblet cells (Figure 4F). This suggested that mitochondrial activity may be a switch in the direction of intestinal stem cell differentiation and the reduction in mitochondrial activity may also be a marker of goblet cell maturation. GSEA and differential analysis indicated that goblet cells in the defunctioned intestine were enriched of gene sets related to oxidative phosphorylation and other stem cell characteristics (Figure 4G and Figure S3D). Collectively, the results indicated that the maturation of goblet cells in the defunctioned intestine may be defective.

**FIGURE 5:**
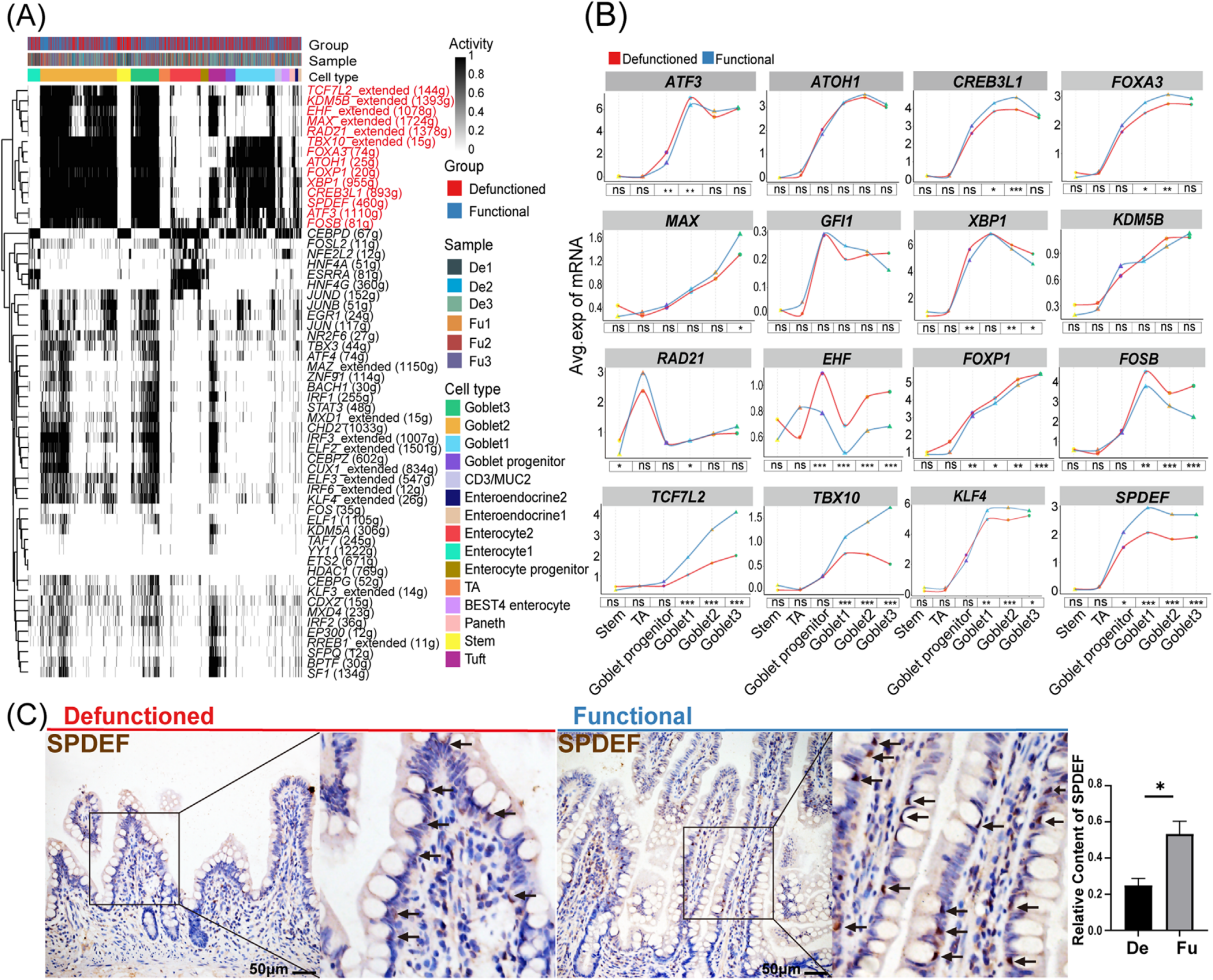
Transcription factor analysis in intestinal goblet cells. (A) Heat map showing the regulon activity scored using the R package AUCell in epithelial cells. Each row represents a regulon, and each column represents a cell. (B) Expression of transcription factors enriched in goblet cells of the functional (Fu) and defunctioned (De) intestines along the developmental trajectory (*p < .05, **p < .01, ***p < .001, ns = not significant, Wilcoxon rank‐sum test). (C) Immunohistochemistry staining showing the expression of SPDEF in goblet cells of the Fu and De intestines (*p < .05, *n* = 5, scale bars = 50 μm, paired Wilcoxon rank‐sum test. The values are presented as mean ± SEM of each group).

We further explored the reasons behind the defect of goblet cell in the defunctioned intestine. We found a significant decrease in goblet cells (stained by AB–PAS) but no significant changes in the number of stem (stained by LGR5 and OLFM4), Paneth (stained by LYZ) or enteroendocrine cells (stained by CHGA) in the defunctioned intestine compared to that in the functional intestine (Figure 4H). This indicated a relative increase in enteroendocrine cells and Paneth cells in the atrophic defunctioned mucosa. Intestinal stem cells differentiate into secretory progenitor cells, which further differentiate into various secretory cells.^42^ Therefore, the differentiation of goblet cells in the defunctioned intestine appears to be hindered. We performed TF analysis (see Section 2) on intestinal epithelial cells. As shown in Figure 5A and Figure S3E, 16 TFs (e.g. *TBX10*, *SPDEF* and *ATOH1*) were relatively enriched in goblet cells. We then performed differential analysis along the developmental trajectory of goblet cells. As expected, the expression of genes that have been reported to be involved in the differentiation and maturation of goblet cells, such as *CREB3L1*,^43^ *FOXA3* ^44^ and especially *SPDEF*,^45^ was significantly reduced in goblet cells of the defunctioned intestine (Figure 5B, Figure S3F). The change in SPDEF protein level was further validated by IHC staining (Figure 5C). In addition, the expression of *FOSB*, *FOXP1* and *EHF* increased, and the expression of *TCF7L2* and *TBX10* decreased in goblet cells of the defunctioned intestine (Figure 5B and Figure S3F). Faecal diversion affected the expression of TFs, such as SPDEF, which may be the main reason for the defective differentiation and maturation in goblet cells of the defunctioned intestine.

### 3.4 Mechanical stimulation from faecal stream may promote differentiation and maturation of intestinal goblet cells through the TRPA1‐ERK pathway

**FIGURE 6:**
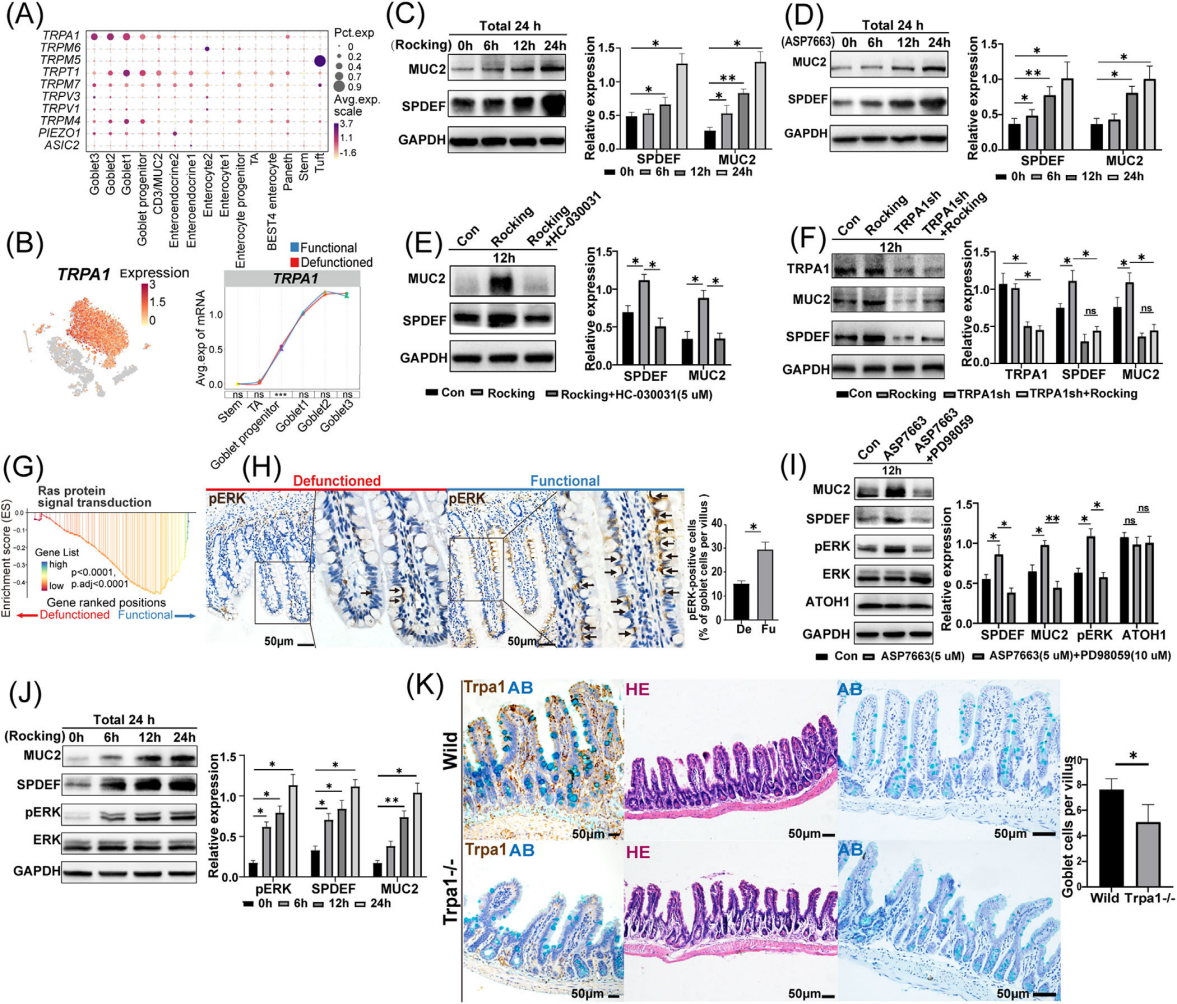
The role of TRPA1 in intestinal goblet cells. (A) Dot plot showing the expression of mechanosensitive ion channels in intestinal epithelial cells. (B) t‐Stochastic neighbor embedding (tSNE) plot showing the expression of *TRPA1* in epithelial cells; the expression of *TRPA1* in goblet cells of the functional and defunctioned intestines (***p < .001; ns = not significant, Wilcoxon rank‐sum test). (C and D) Mechanical stimulation and TRPA1‐selective agonist (ASP7663, 5 μM) promoted the expression of SPDEF and MUC2 (*n* = 3). All cells were cultured for 24 h and treated with a rocking board (Rocking) for mechanical stimulation or ASP7663 for 6, 12 and 24 h, respectively. (E) Treatment with TRPA1‐selective inhibitor (HC‐030031, 5 μM) blocked the expression of SPDEF and MUC2 induced by mechanical stimulation (*n* = 3). (F) Blocking the expression of TRPA1 inhibited the expression of SPDEF and MUC2 induced by mechanical stimulation (*n* = 3). (G) Gene set enrichment analysis showed enriched RAS signal in goblet cells of the functional intestine. (H) Immunohistochemistry staining of pERK in functional and defunctioned intestines (*p < .05, *n* = 5, scale bars = 50 μm, paired Wilcoxon rank‐sum test). (I) Treatment with ASP7663 (5 μM) promoted the expression of pERK and treatment with ERK selective inhibitor (PD98059, 10 μM) inhibited the expression of SPDEF and MUC2 induced by ASP7663; the expression of ATOH1 was not affected (*n* = 3). (J) Mechanical stimulation promoted the expression of SPDEF, MUC2 and pERK (*n* = 3). All cells were cultured for 24 h, and cells were mechanically stimulated using a Rocking for 6, 12 and 24 h, respectively. (K) Immunohistochemistry staining for Trpa1, HE staining and alcian blue (AB) staining of intestine in wild‐type and Trpa1^−/−^ mice (*p < .05, *n* = 5, scale bars = 50 μm, Wilcoxon rank‐sum test). (C–F) and (I–J); *p < .05, **p < .01, ns = not significant, unpaired *t*‐test. All values are presented as mean ± SEM of each group. Con, control.

GSVA indicated that the goblet cells have a high score for sensing mechanical stimulation (Figure S3G). Mechanical stimulation, as a main factor involved in the loss of defunctioned intestine, may play an important role in the differentiation and maturation of intestinal goblet cells. To further investigate this, we explored the expression of mechanosensitive ion channels, such as the TRP and PIEZO channels, in epithelial cells (Figure 6A). We found that goblet cells significantly expressed *TRPA1*, and its expression gradually increased with the maturity of goblet cells; however, it was not reduced in goblet cells of the defunctioned intestine (Figure 6A,B). Previous studies have shown that the hyperplasia of goblet cells is reduced in the airways of the lungs of Trpa1^−/−^ mice.^46^ To verify the role of TRPA1 in intestinal goblet cells, we selected LS174T cells, a common cell line used for investigating goblet cell function, as the object of in vitro study and simulated mechanical stimulation with a rocking board, according to the study by Xu et al.^47^ We observed that mechanical stimulation promoted the expression of SPDEF and MUC2, and that TRPA1 agonists (ASP7663) had similar effects (Figure 6C,D). By contrast, TRPA1‐selective antagonist (HC‐030031) reversed the effects of mechanical stimulation (Figure 6E). Moreover, we customized shRNA for the *TRPA1* gene sequence and successfully transfected LS174T cells, which reduced TRPA1 expression and reversed the effects of mechanical stimulation (Figure 6F).

Subsequently, we explored the possible downstream pathways following the activation of TRPA1 by mechanical stimulation. GSEA indicated that the RAS signalling pathway was notably down‐regulated in goblet cells of the defunctioned intestine (Figure 6G). ERK is one of the major downstream targets of the RAS pathway, and influx of Ca^2+^ ions mediated by TRPA1 can also activate ERK.^48^ Therefore, we speculated that mechanical stimulation activated TRPA1, which led to inwards calcium flow and further activation of ERK, ultimately promoting the expression of SPDEF and MUC2. To confirm this hypothesis, we performed in vitro experiments and found that mechanical stimulation and selective activation of TRPA1 promoted ERK phosphorylation, whereas ERK inhibition blocked TRPA1‐induced expression of SPDEF and MUC2 in LS174T cells (Figure 6I,J). Furthermore, IHC staining displayed that pERK‐positive goblet cells in the defunctioned intestine were significantly reduced (Figure 6H). ATOH1 is an upstream transcription factor of SPDEF, which is required for the differentiation of all secretory cells.^49^ However, activation of TRPA1 and inhibition of ERK did not alter ATOH1 expression (Figure 6I), which suggested that mechanical stimulation‐induced SPDEF expression was not due to the expression of ATOH1 and that there were other mechanisms downstream of pERK. In addition, we also compared the ileal mucosa of Trpa1^−/−^ mice with that of wild‐type mice and found the number of goblet cells in Trpa1^−/−^ mice were significantly reduced (Figure 6K). Mechanical stimulation promotes SPDEF expression in cell lines via TRPA1‐ERK, which suggested that mechanical stimulation from faecal stream may also promote SPDEF expression in goblet cells.

### 3.5 Ileal faecal diversion promotes fibrosis in the defunctioned intestine

**FIGURE 7:**
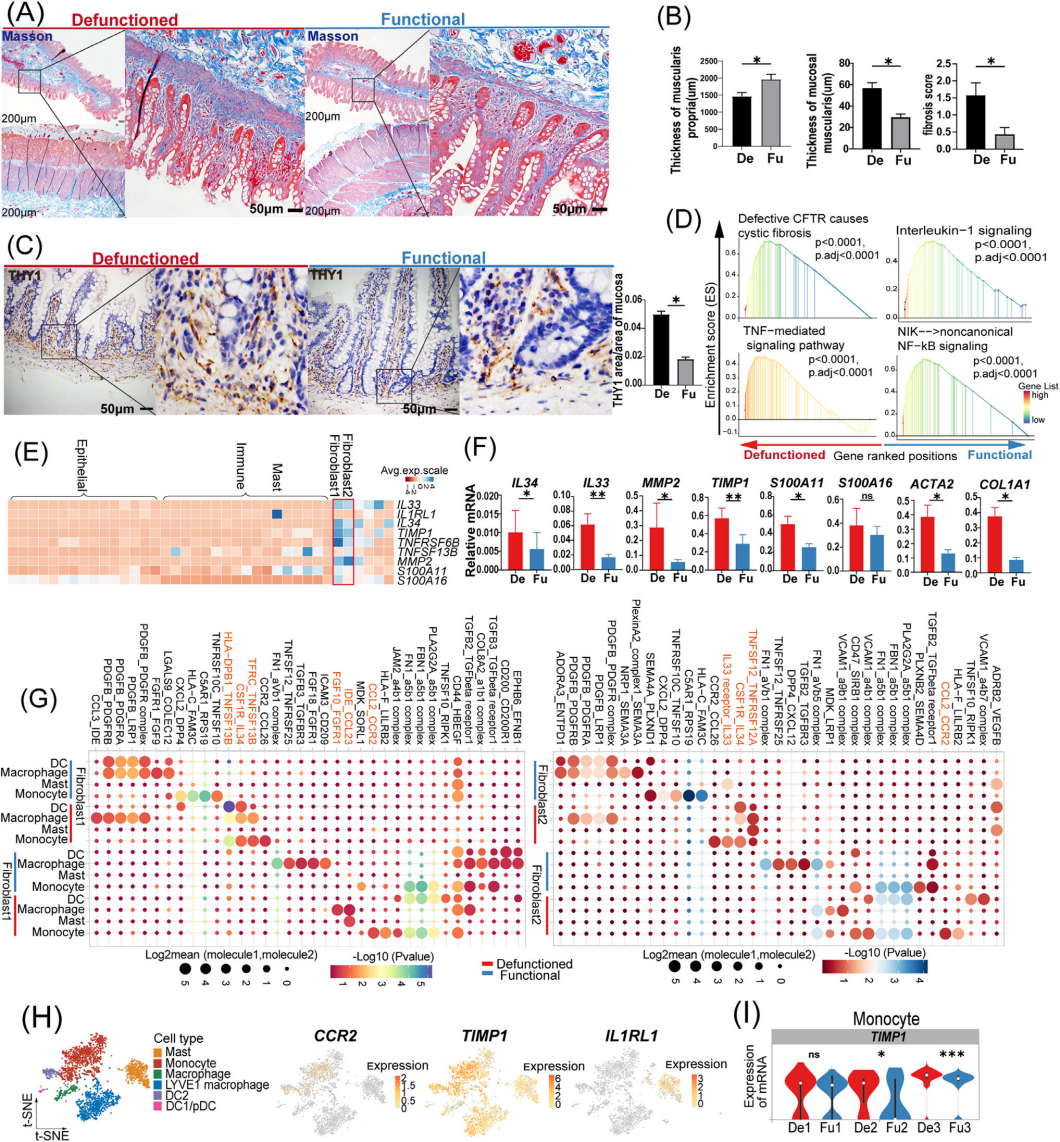
Changes in fibroblasts of the defunctioned (De) intestine and the interaction between fibroblasts and myeloid‐mast cells. (A) Masson's trichrome staining of the functional (Fu) and De intestines (scale bars = 200 or 50 μm). (B) Thickness of the mucosal muscularis and muscularis propria and fibrosis scores in the Fu and De intestines (*p < .05, *n* = 5). (C) Immunohistochemistry staining of THY1 showing fibroblasts in the Fu and De intestines (*p < .05, *n* = 5, scale bars = 50 μm). (D) Gene set enrichment analysis showing some enriched inflammatory and fibrosis‐related signalling pathways in fibroblasts of the De intestine. (E) Heat map showing the expression of fibrosis‐related genes in all cell types. (F) Relative mRNA expression of the fibrosis‐related genes in fibroblasts of the Fu and De intestines was confirmed by quantitative PCR (*p < .05, **p < .01, ns = not significant, *n* = 6 or 7). (G) Dot plot showing ligand–receptor interactions between fibroblasts and myeloid‐mast cells in the Fu and De intestines as predicted using CellPhoneDB analysis. Colour indicates permutation *p*‐value, and point size indicates the scaled mean expression level of ligand and receptor. (H) t‐Stochastic neighbor embedding (tSNE) plot showing the expression of *CCR2*, *TIMP1* and *IL1RL1* in myeloid‐mast cells. (I) Violin plots showing the expression of *TIMP1* in monocytes of the Fu and De intestines (*p < .05, ***p < .001, ns = not significant, Wilcoxon rank‐sum test). (B, C, F) Paired Wilcoxon rank‐sum test. All values are presented as mean ± SEM of each group.

Prolonged faecal diversion often leads to fibrosis in the defunctioned intestine.^50^ Therefore, we used Masson's trichrome staining to assess fibrosis in the defunctioned intestine (Figure 7A). We found more collagen fibrils deposited in the mucosal layer and more hypertrophy of the mucosal muscularis, in contrast to the previously reported atrophy of the muscularis propria in the defunctioned intestine (Figure 7A,B), which indicated that fibrosis in the defunctioned intestine may be more limited to the mucosal layer. Fibroblasts are important components of mesenchymal cells and play an important role in intestinal fibrosis.^51^ There were more fibroblasts in the defunctioned intestine, which was verified by IHC staining (Figure 7C). According to our data, GSEA also showed that fibroblasts in the defunctioned intestine were enriched of inflammatory and fibrosis‐related signalling pathways (e.g. defective CFTR causes cystic fibrosis and TNF‐mediated signalling pathway) (Figure 7D). In addition, intestinal fibroblasts notably expressed some pro‐fibrotic factors, such as *IL‐33*, *IL‐34*, *TIMP1*, *MMP2*, *S100A11* and *S100A16* (Figure 7E), ^51^, ^52^, ^53^, ^54^ and subsequent quantitative PCR analysis showed that their expression was significantly increased in fibroblasts of the defunctioned intestine, except for *S100A16* (Figure 7F). The expression of *COL1A1* and *ACTA2* in fibroblasts of the defunctioned intestine was also significantly increased (Figure 7F). These results suggested that fibroblasts in the defunctioned intestine exhibited several pro‐fibrotic characteristics.

Intestinal fibrosis is a complex process involving interactions among different cell types. Thus, we used CellphoneDB to construct a communication network between fibroblasts and other cell types in the functional and defunctioned intestines, respectively. As shown in Figure S4A,B, there are abundant cellular interactions between fibroblasts and other cells. Among them, we observed that some ligand–receptor pairs (e.g. *CCL2*_*CCR2*, *CSF1R*_*IL34*, *HLA−DPB1*_*TNFSF13B*, *IL33* receptor_*IL33* and *TNFSF12*_*TNFRSF12A*) appeared more frequently between fibroblasts and myeloid cells in the defunctioned intestine (Figure 7G). The CCL2/CCR2 axis is involved in fibrotic diseases. Fibroblasts recruit CCR2^+^ monocytes by expressing CCL2, and monocytes express TIMP1 and promote intestinal fibrosis.^55^ We also found higher expression of *TIMP1* in monocytes of the defunctioned intestine (Figure 7H,I). Furthermore, *IL34* and *IL33* promote M2 polarization of macrophages, which is closely related to fibrosis.^56^, ^57^, ^58^ According to GSEA, macrophages of the defunctioned intestine tended to show M2 activation (IL‐4 and IL‐13 signalling), whereas macrophages of the functional intestine were enriched in M1 activation (INF‐γ‐mediated signalling pathway) (Figure S4C,D). In addition, mast cells communicated with fibroblasts with a particularly high expression of the *IL33* receptor *IL1RL1* (Figure 7E,H), and GSEA displayed pro‐inflammatory and pro‐fibrotic properties in mast cells of the defunctioned intestine (Figure S4E). There appeared to be a pro‐fibrotic microenvironment in the defunctioned intestine, but further experimental validation is needed.

Intestinal fibroblasts also play an important role in the renewal, proliferation and differentiation of intestinal epithelial cells.^59^ According to our data, the analysis of the interaction between intestinal fibroblasts and epithelial cells showed that some ligand–receptor pairs (e.g. *FGFR2*_*FGF9*/*FGF18*, *BMPR1A*/*BMPR2*_*BMP7*/*BMP4* and *WNT5A*_*PTPRK/FZD5*) were less abundant in the defunctioned intestine (Figure S4F,G). This may be related to atrophy and defects in the epithelium of the defunctioned intestine because *FGF9*, *BMP4* and *WNT5A* have been reported to regulate intestinal development.^60^, ^61^, ^62^

### 3.6 Monocytes may be important targets for faecal diversion to alleviate CD

**FIGURE 8:**
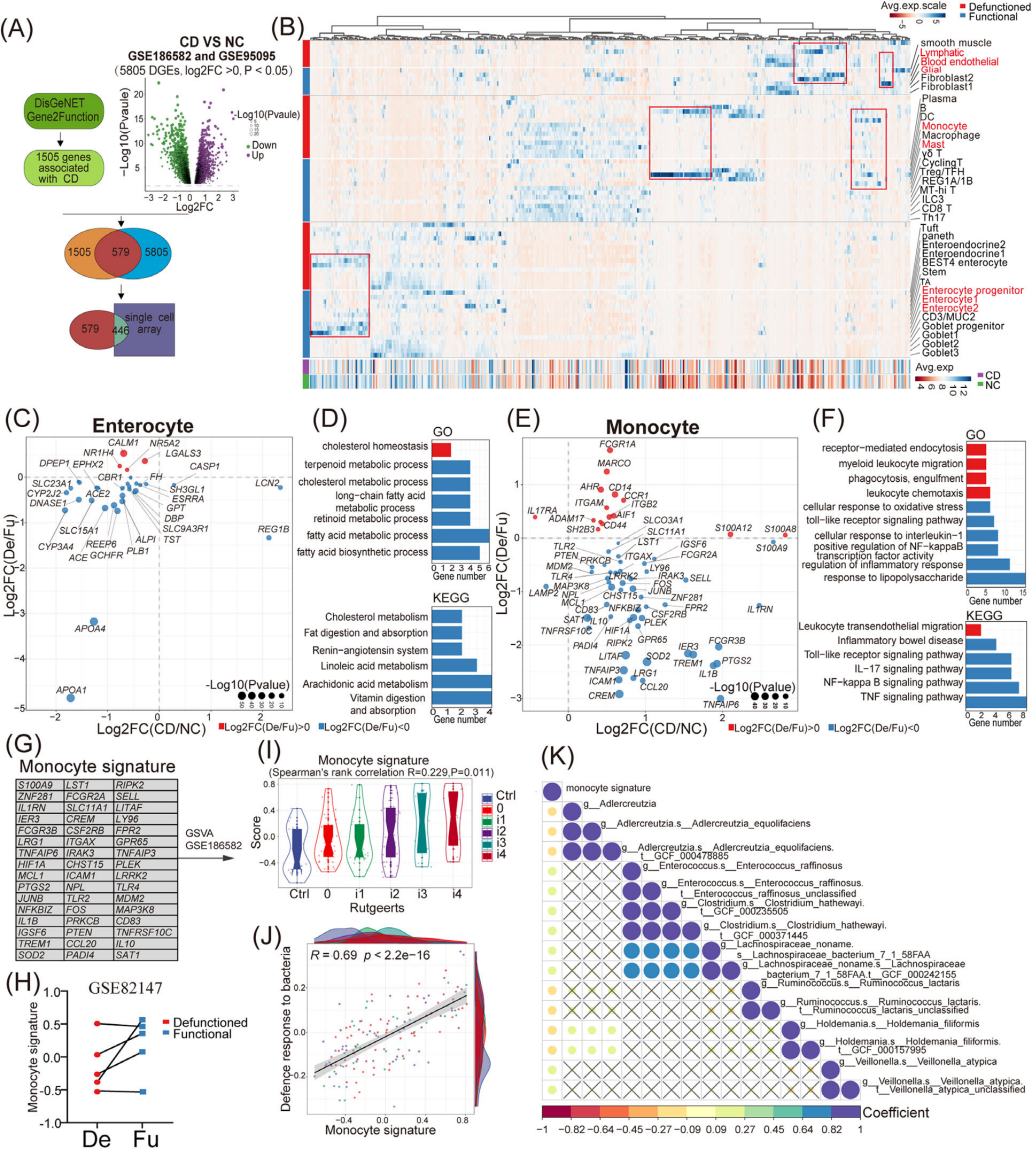
Joint analysis of our single‐cell data and Crohn's disease (CD)‐related public databases. (A) Collection and selection of genes significantly related to CD. (B) Heat map showing the expression of 446 CD‐related genes in all cell types of the defunctioned (De) and functional (Fu) intestines and in CD and normal tissues. Data on CD and normal tissues from the GSE186582 and GSE95095 datasets. (C and E) Dot plot showing the log2 fold change in the expression of genes significantly altered in enterocytes or monocytes of the De intestine and in CD tissue (*p* < .05). Colours indicate the up‐regulation (red) or down‐regulation (blue) in gene expression in the De intestine. (D and F) Gene ontology and Kyoto Encyclopedia of Genes and Genomes enrichment analyses of the genes showed in (C and E). Colours indicate the same meaning. (G) A number of 48 non‐overlapping signature genes from faecal diversion‐induced monocyte alterations as a monocyte signature. These genes were down‐regulated in monocytes of the De intestine and up‐regulated in CD. (H) Monocyte signature scored per sample using gene set variation analysis (GSVA) in dataset GSE82147 with paired Fu and De intestines. (I and J) Correlation between monocyte signature score and Rutgeerts score or the signature (defence response to bacteria) in dataset GSE186582. Rutgeerts scores are used to assess the extent of intestinal lesions in CD patients. The scores of the signatures were scored using GSVA (Spearman's rank correlation, *p* < .05). (K) Correlation between monocyte signature score scored using GSVA and microbial taxa in CD datasets (HMP2) with paired tissue sequencing and metagenomics sequencing (Spearman's rank correlation, *p* < .05). Ctrl, control; NC, normal control.

Ileal faecal diversion plays an important role in CD treatment. We performed a joint analysis of our data and CD‐related public databases to explore the mechanism of faecal diversion in alleviating CD. We obtained 579 significantly related CD genes and mapped them to our data (Figure 8A, see Section 2). In the context of 446 CD‐related genes (Table S10), faecal diversion led to alterations in the expression patterns of mast cells, glial cells, blood endothelial cells, lymphatic endothelial cells and, in particular, enterocytes and monocytes (Figure 8B,C,E and Figure S5A). Compared to those in normal or functional intestines, changes in enterocytes due to faecal diversion, such as reduced *APOA1*and *APOA4* expression, were similar to those caused by CD (Figure 8C), which were mainly related to metabolism and absorption (e.g. cholesterol metabolism) according to GO and KEGG enrichment (Figure 8D). By contrast, faecal diversion‐induced changes in monocytes, such as reduced *IL1B*, *IL1RN*, *TLR4* and *PTGS2* expression, were almost opposite to those caused by CD (Figure 8E), which were mainly involved in inflammation and bacterial responses (e.g. TNF signalling pathway and response to lipopolysaccharide) (Figure 8F). CD is characterized by expanded subpopulations of inflammatory monocytes, which exacerbate inflammatory damage.^63^, ^64^ Consistent with this, we extracted 48 non‐overlapping signature genes from faecal diversion‐induced monocyte alterations as a monocyte signature and performed cell scoring with Seurat in a single‐cell dataset of CD (GSE134809) from Martin et al.^18^ (Figure 8G and Figure S5B). We identified two monocyte clusters with high monocyte signature scores, including monocytes (marked by *FCN1*, *S100A9* and *S100A8*) and inflammatory monocytes/macrophages (marked by *S100A9*, *S100A8*, *IL1B* and *C1QA*), which were mainly found in CD tissues (Figure S5C–F). The monocyte signature scores in the CD tissues were higher than those in the normal tissues (Figure S5G). Collectively, these findings indicated that faecal diversion may alleviate CD by reversing the inflammatory changes in CD‐associated monocytes.

Considering that only approximately 50%–60% of patients with CD have a clinical response to faecal diversion,^65^, ^66^ we further evaluated the monocyte signature scores in some public datasets using GSVA. We found that a portion of the defunctioned intestines had a reduced score in the GSE82147^16^ dataset, with paired functional and defunctioned intestine data (Figure 8H). In the CD dataset GSE186582,^67^ the monocyte signature scores were positively correlated with the Rutgeerts scores (Figure 8I), suggesting that scores of monocyte signature correlated with the severity of CD, and patients with high scores may be more suitable for faecal diversion. To further evaluate the bacterial responses of monocytes, the scores of a signature (defence response to bacteria) obtained from the GO database were evaluated, and they were significantly positively correlated with the monocyte signature scores (Figure 8J). Furthermore, in CD datasets (HMP2) with paired tissue sequencing and microbial sequencing from Lloyd‐Price et al.,^68^ we observed that monocyte signature scores were positively correlated with the abundance of some microbes, such as *Enterococcus raffinosus*, *Clostridium hathewayi* and *Veillonella atypica* (Figure 8K and Figure S5H), which may cause abnormal monocyte activation.

## 4 DISCUSSION

Ileal faecal diversion deprives most luminal stimulation in the distal intestine. As a result, without scouring of the luminal contents, the distal intestine forms a novel temporary or permanent intestinal ecology after long‐term adaptation. This unique model provides a great opportunity to investigate the underlying functions of luminal stimulation on intestinal pathophysiology. Therefore, we surveyed, for the first time, the gene expression profiles of functional and defunctioned intestines using scRNA‐seq to outline the cellular and molecular changes behind faecal diversion. Our data demonstrated new functional changes in the defunctioned intestine and provided clues regarding the significant role of mechanical stimulation in the differentiation and maturation of goblet cells through the TRPA1‐ERK pathway. Additionally, joint analysis of monocyte signatures with ileal faecal diversion and CD data revealed that monocytes are the possible key targets of faecal diversion to alleviate CD. These results elucidate the effects of faecal diversion and provide a basis for the further investigation of the pathogenesis and treatment of CD.

Previous studies have shown that starvation, fasting and total parenteral nutrition result in mucosal atrophy characterized by morphological changes and diminished intestinal function, including decreased surface area, crypt depth, villous height and epithelial cell number.^69^ However, patients with ileal faecal diversion can receive adequate enteral nutrition, and the defunctioned intestinal mucosa can also receive enteral nutrition from functional intestinal segments through the mesentery. Atrophy of the defunctioned intestinal mucosa was perhaps an adaptation mainly due to the lack of direct interaction of intestinal contents with the intestinal mucosa, such as mechanical stimulation. Recent studies have demonstrated that mechanical stimulation promotes the development and maturation of human intestinal organoids in vivo, including increased villus length and crypt depth.^70^ We also found that the epithelium in the defunctioned intestine tended to be immature, with more transcriptional characteristics of stem or progenitor cells compared to the functional epithelium. Therefore, the absence of mechanical stimulation may be an important cause of defunctioned intestinal mucosal atrophy, and supplemental mechanical stimulation before the reversal of faecal diversion may reverse the atrophy of the defunctioned intestinal mucosa and accelerate the recovery of patients. Furthermore, the significant reduction of NTS expression in the defunctioned intestine may also contribute to intestinal mucosal atrophy as NTS has recently been reported to regulate intestinal epithelial proliferation and stem cell function.^71^

Associated with immaturity of the epithelium in the defunctioned intestine, we found a defective mechanical and mucus barrier, as well as defective metabolic absorption in the defunctioned intestine, indicating that the defunctioned intestine has a weakened ability to defend itself against pathogens. Meanwhile, faecal diversion disrupts the continuity of the intestine, which deprives the source of nutrition for microbes in the defunctioned intestine and allows oxygen to enter the intestinal lumen, leading to a reduction in microbial abundance and disruption of microbial composition.^50^, ^72^ This may stimulate the growth of pathogenic microbes. Additionally, significantly reduced T cells and IgA^+^ B cells indicate reduced adaptive immunity.^73^, ^74^ Consequently, the defunctioned intestine has a high risk of infection. Nevertheless, our data showed that the antimicrobial peptides of the epithelium in the defunctioned intestine were significantly increased, compensating for the defective function of the intestinal barrier. Despite this, with the reversal of faecal diversion, the rapid change in the environment of the intestinal cavity may stimulate the breeding of some conditional pathogens, such as *Clostridium difficile*, which increases the risk of infection and anastomotic leakage.^75^ Therefore, the use of local antibiotics before the reversal of faecal diversion may be necessary.^76^

In addition to immature goblet cells, we also demonstrated defective differentiation of goblet cells caused by the absence of mechanical stimulation in the defunctioned intestine. He et al. found that mechanical stimulation regulates the differentiation of intestinal stem cells via the PIEZO1 channel in *Drosophila*.^77^ A recent study pointed out that mechanical stimulation enhances the expression of MUC2 by activating the PIEZO1 channel in the LS174T cell line.^47^ Distinguished from it, in our data, we found that mechanical stimulation also promoted the expression of SPDEF via the TRPA1 channel in the LS174T cell line and normal goblet cells exhibited high expression of TRPA1, and there were reduced intestinal goblet cells in Trpa1^−/−^ mice. Therefore, TRPA1 might have a significant role in the differentiation and maturation of intestinal goblet cells. The expression of TRPA1 gradually increased with the maturation of goblet cells, suggesting that mechanical stimulation becomes increasingly important for goblet cell differentiation and maturation as stem cells leave the intestinal crypts. We also found that TRPA1 regulated the expression of SPDEF through the ERK pathway but did not affect the expression of ATOH1, which is an upstream transcription factor of SPDEF. The downstream pathway of TRPA1 requires further investigation. Defects in goblet cells in the defunctioned intestine are a risk factor for intestinal obstruction after the reversal of faecal diversion because mucus also plays a role in lubrication.

Analysis of the stromal changes in the defunctioned intestine revealed the fibrosis of the mucosal layer, and previously reported atrophy of the muscularis propria was observed. Fibrosis of the mucosal layer revealed chronic inflammation in the defunctioned intestine, which may be associated with the dysbiosis of the intestinal microbes and lead to intestinal stenosis, stiffening and motility disorders.^78^ The atrophy of the muscularis propria may be due to the absence of mechanical stimulation, leading to a loss of intestinal motility.^70^ Dysbiosis of microbes in the defunctioned intestine also results in decline in intestinal motility, which has been reviewed previously.^74^ These are risk factors for slow recovery and complications after reversal of faecal diversion, which need to be addressed. Consistent with fibrosis, we found a significant increase in fibroblast counts of the defunctioned intestine that exhibited some pro‐fibrotic characteristics, such as increased expression of *IL33*, *IL34*, *TIMP1* and *MMP2*. IL33 and IL34 were not only able to directly promote collagen production by fibroblasts^79^, ^80^ but also induce M2 polarization of macrophages, which was observed in the defunctioned intestine according to GSEA and may promote intestinal fibrosis. IL33 also acted on monocytes, and the expression of *TIMP1* in monocytes of the defunctioned intestine was increased. The defunctioned intestine may have an imbalance between tissue inhibitors of metalloproteinases and matrix metalloproteinases, which may be induced by increased IL33 expression.^81^ Moreover, mast cells in the defunctioned intestine exhibited pro‐inflammatory and pro‐fibrotic properties, which may also be related to IL33. Altogether, IL33 may be a key factor of fibrosis in the defunctioned intestine.

Although the intestinal changes caused by ileal faecal diversion have a negative side, they also have a positive side, such as relieving CD. We demonstrated that monocytes may be key targets for faecal diversion to alleviate CD. Monocytes, as an important component of innate immunity, play a crucial role in CD pathophysiology, according to recent evidence. On the one hand, monocytes in CD have immune deficiencies such as lack of NOD2‐mediated immune regulation and failure to induce Paneth cell defensins.^63^, ^82^ On the other hand, monocytes in CD are abnormally stimulated and show strong pro‐inflammatory properties such as promoting Th17/Th1 responses through IL‐1β, inducing barrier defects by IL1β and IL8 and regulating monocyte IL‐23 production.^83^, ^84^, ^85^ Our analysis suggested that faecal diversion may attenuate inflammatory and microbial responses in monocytes, thus relieving CD. However, only about half of patients with CD respond clinically to faecal diversion. This was consistent with the non‐reduction of monocyte scores in part of the defunctioned intestines in our analysis. In addition, Spivak et al. found that the clinical response of patients with CD to faecal diversion was highly correlated with the expression of I2 antibodies against *Pseudomonas fluorescens*.^86^ *P. fluorescens* activates monocytes and disrupts intestinal barrier function.^87^ These results indicate that the differences in clinical responses may be related to the composition of the intestinal microbiome in patients with CD. Our analysis also showed that monocyte signature scores were positively correlated with the abundance of some microbes, such as *E. raffinosus*, *C. hathewayi* and *V. atypica* in patients with CD. These microbes may cause abnormal activation of monocytes and may be used as predictive targets for clinical response but require further exploration.

In summary, faecal diversion provides a window for understanding the physiological and pathological roles of the faecal stream in the intestine. Defects in the intestinal epithelium and enhanced fibrosis further indicated that pre‐stimulation of the defunctioned intestine before the reversal of faecal diversion is meaningful and necessary. Exploring the underlying mechanisms will provide innovative ideas and guidance for pre‐stimulation methods, but this will require experimental and long‐term clinical validation. The combined analysis of our data and CD data suggested that it may be possible to target monocytes and thereby treat CD. However, our study also has some limitations. First, many inferences in our study lacked experimental validation. Second, our limited sample size may result in bias in our results; last, we lacked biological samples from healthy people to exclude the possible influence of other factors.

## CONFLICT OF INTEREST STATEMENT

The authors declare that they have no conflicts of interest.

## Supporting information

Supporting Information

Supporting Information

Supporting Information

Supporting Information

Supporting Information

Supporting Information

Supporting Information

Supporting Information

Supporting Information

Supporting Information

## Data Availability

The data that support the findings of this study are available in the National Genomics Data Center (NGDC, https://ngdc.cncb.ac.cn/) with accession number HRA004177. [GSE186582] Ngollo M, Perez K, Hammoudi N, Gorelik Y et al; 2022; Expression data from intestinal mucosa of patients with Crohn disease; GEO Data Sets; https://www.ncbi.nlm.nih.gov/geo/query/acc.cgi?acc=GSE186582. [GSE95095] Zhao X; 2019; Differential Gene Expression between involved and uninvolved sites of Crohn's disease: Insights into a Distinctive Pathogenesis Profile; GEO Data Sets; https://www.ncbi.nlm.nih.gov/geo/query/acc.cgi?acc=GSE95095. [GSE134809] Martin JC, Chang C, Boschetti G, Ungaro R et al; 2019; Single‐cell analysis of Crohn's disease lesions identifies a pathogenic cellular module associated with resistance to anti‐TNF therapy; https://www.ncbi.nlm.nih.gov/geo/query/acc.cgi?acc=GSE134809. [HMP2] Lloyd‐Price J, Arze C, Ananthakrishnan AN, Schirmer M et al; 2019; Longitudinal Multi'omics of the Human Microbiome in Inflammatory Bowel Disease; the Inflammatory Bowel Disease Multi'omics Database; http://ibdmdb.org. [GSE82147] Wieck M, Grikscheit T, Grubbs B, Thornton M et al; 2016; Genome wide impact of loss of mechanoluminal stimulation on neonatal intestine; GEO Data Sets; https://www.ncbi.nlm.nih.gov/geo/query/acc.cgi?acc=GSE82147.
