# Supplementary figures and images for: Dissecting the effect of ileal faecal diversion on the intestine using single‐cell sequencing
Source: Clin Transl Med. 2023 Jul 3;13(7):e1321. doi: 10.1002/ctm2.1321 (PMC10318127; doi:10.1002/ctm2.1321)

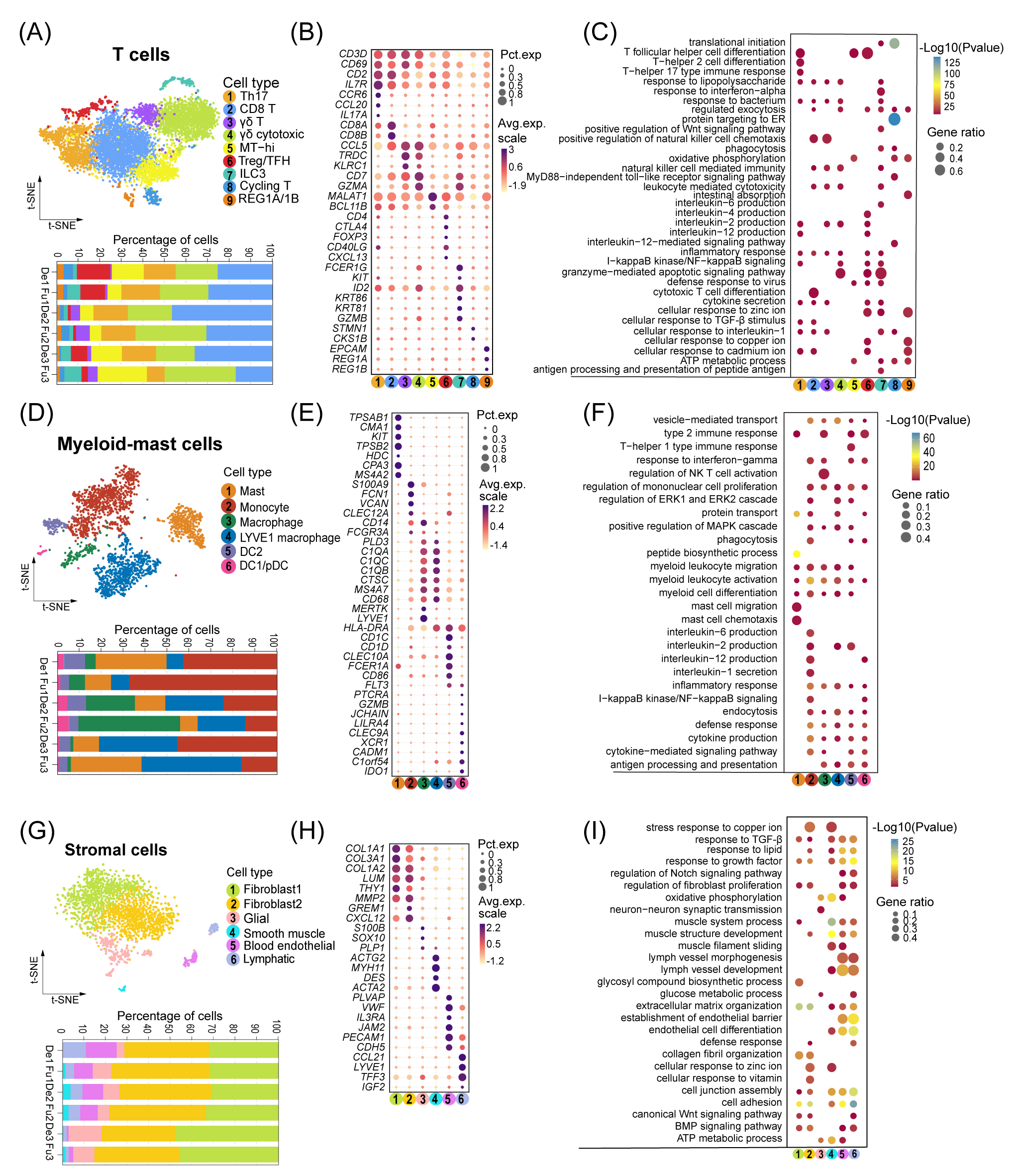

Supplement: Supplementary file 1 — Supporting Information [file CTM2-13-e1321-s003.tif]

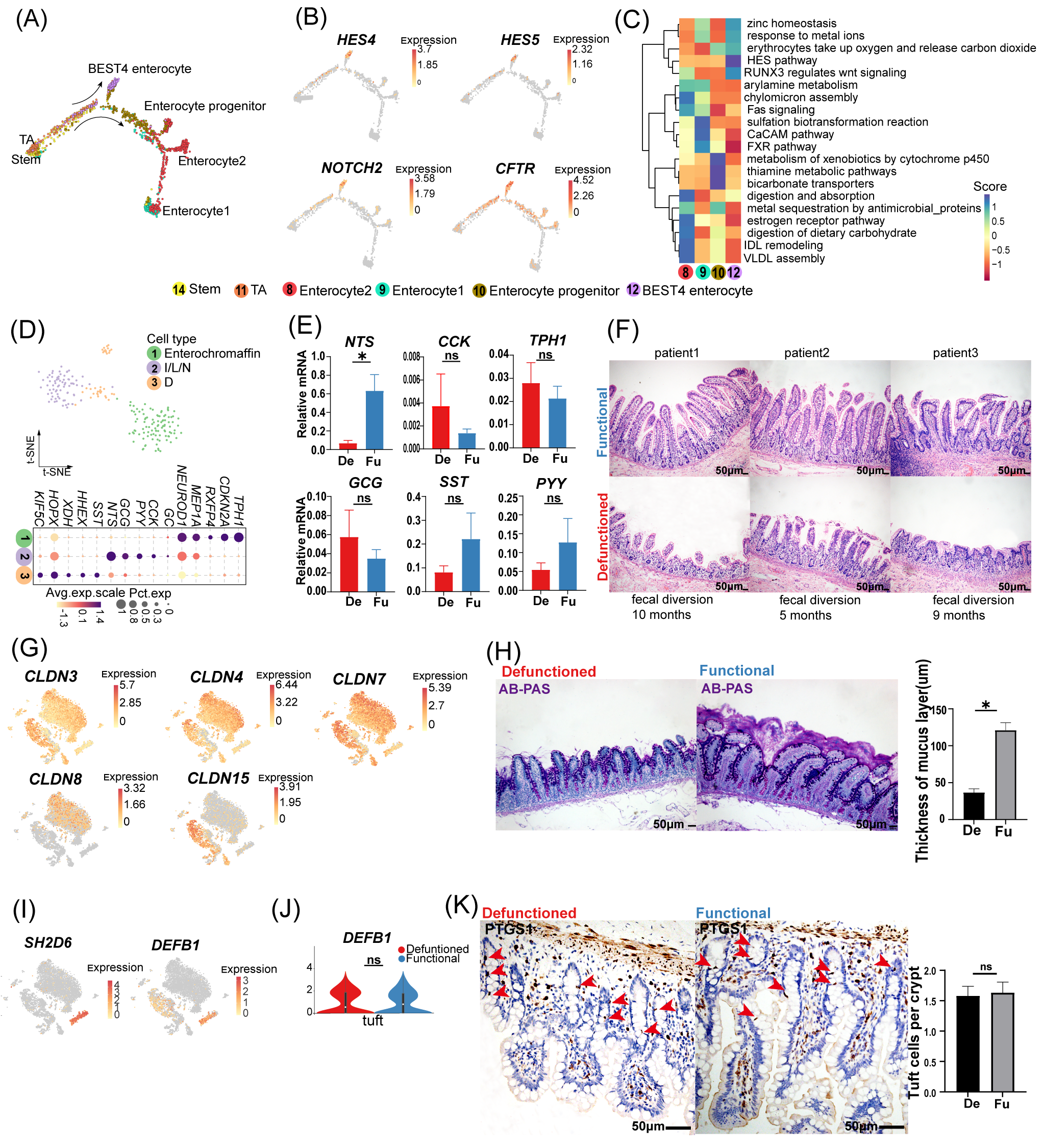

Supplement: Supplementary file 2 — Supporting Information [file CTM2-13-e1321-s006.tif]

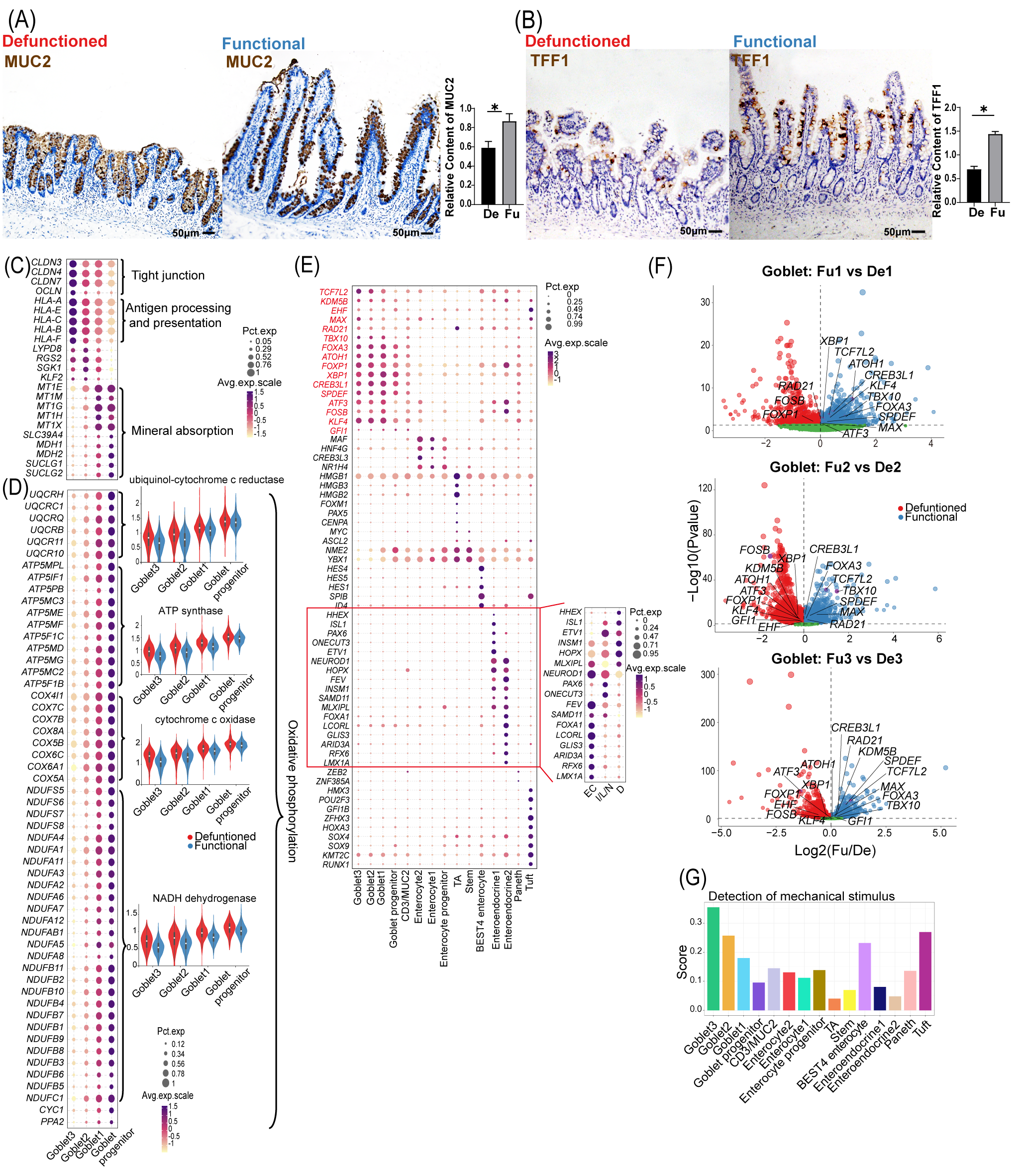

Supplement: Supplementary file 3 — Supporting Information [file CTM2-13-e1321-s001.tif]

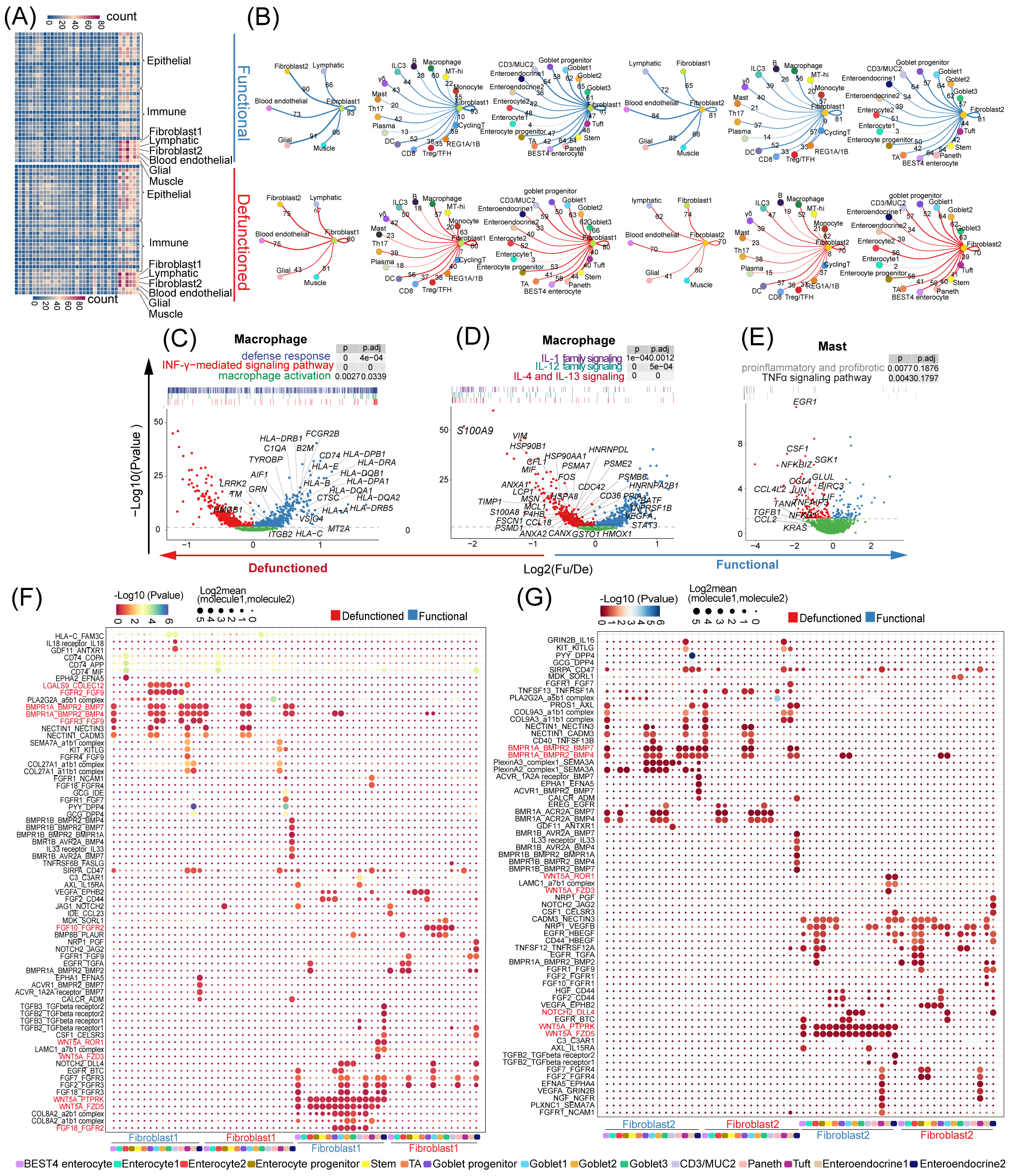

Supplement: Supplementary file 4 — Supporting Information [file CTM2-13-e1321-s004.tif]

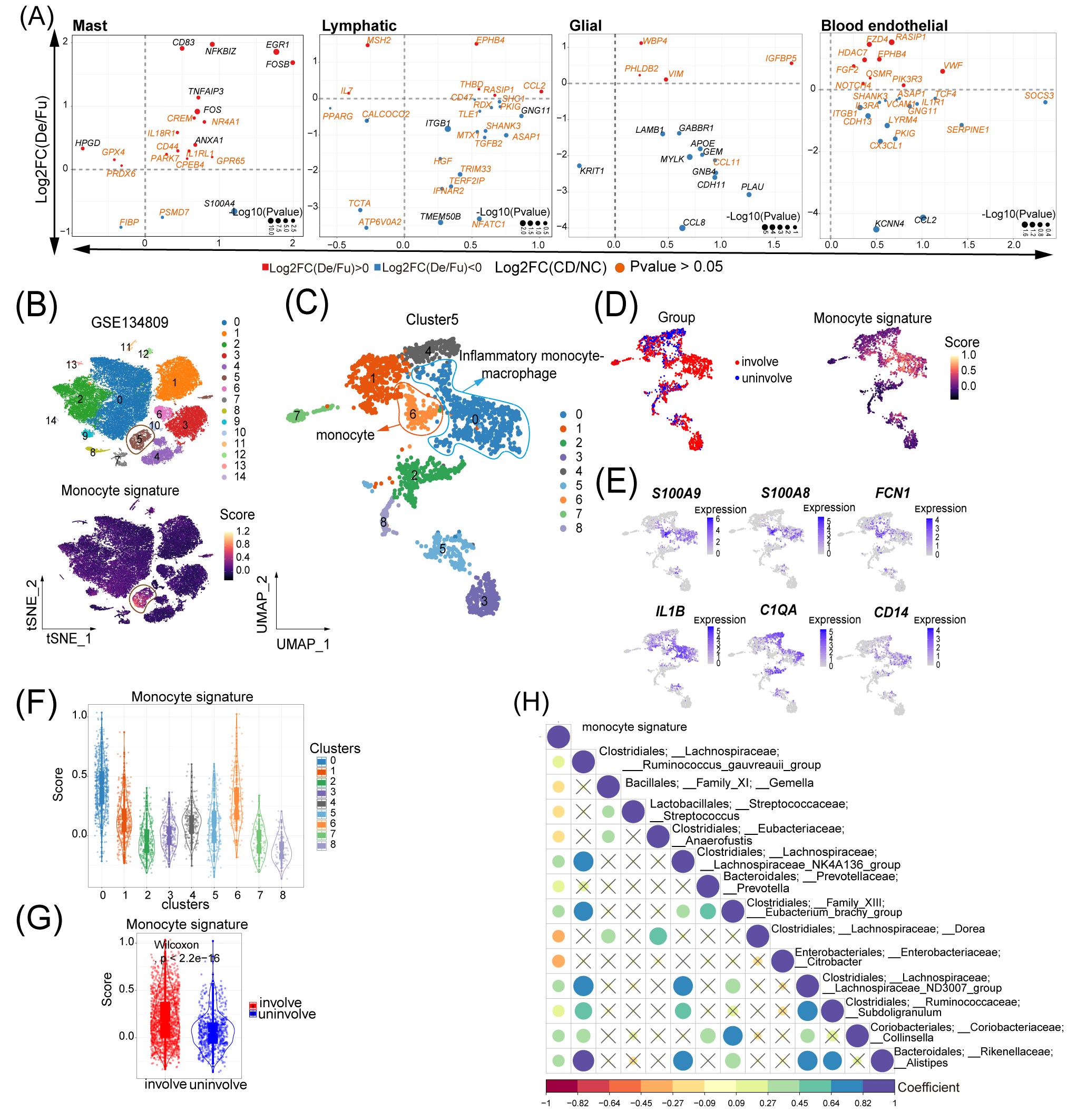

Supplement: Supplementary file 5 — Supporting Information [file CTM2-13-e1321-s002.tif]
