# Supplementary material for: Dissecting the effect of ileal faecal diversion on the intestine using single‐cell sequencing
Source: Clin Transl Med. 2023 Jul 3;13(7):e1321. doi: 10.1002/ctm2.1321 (PMC10318127; doi:10.1002/ctm2.1321)
Supplement: Supplementary file 10 — Supporting Information [file CTM2-13-e1321-s007.docx]

**Supplemental legends**

**FIGURE S1** Classification and functional annotation of T cells, myeloid-mast cells, and stromal cells. (A) tSNE plot showing nine subsets of T cells; the proportion of each subset in T cells of the functional and defunctioned intestines. (B) Dot plot showing the expression of significant marker genes in nine subsets of T cells. (C) GO enrichment of marker genes (log2FC > 1, *P* < 0.05, Wilcoxon rank-sum test) in each subset of T cells. (D) tSNE plot showing six subsets of myeloid-mast cells; the proportion of each subset in myeloid-mast cells of the functional and defunctioned intestines. (E) Dot plot showing the expression of significant marker genes in six subsets of myeloid-mast cells. (F) GO enrichment of marker genes (log2FC > 1, *P* < 0.05, Wilcoxon rank-sum test) in each subset of myeloid-mast cells. (G) tSNE plot showing six subsets of stromal cells; the proportion of each subset in stromal cells of the functional and defunctioned intestines. (H) Dot plot showing the expression of significant marker genes in six subsets of stromal cells. (I) GO enrichment of marker genes (log2FC > 1, *P* < 0.05, Wilcoxon rank-sum test) in each subset of stromal cells. GO, Gene Ontology; De, Defunctioned; Fu, Functional.

**FIGURE S2** Classification and functional annotation of epithelial cells, differences in epithelial cells between the functional and defunctioned intestines. (A) Pseudotime analysis showing different developmental trajectories of enterocytes1, enterocytes2 and BEST4 enterocytes. (B) BEST4 enterocytes show high expression of *HES4*, *HES5*, *NOTH2* and *CFTR*. (C) Differences in pathway activities scored per cell by gene set variation analysis in enterocytes1, enterocytes2, BEST4 enterocytes and enterocyte progenitors. The scores of pathways are normalized. (D) tSNE plot showing three subsets of enteroendocrine cells and dot plot showing expression of significant marker genes in three subsets of enteroendocrine cells. (E) Relative mRNA expression of hormones in epithelial cells of the functional and defunctioned intestines was confirmed by quantitative PCR (**P* < 0.05, ns = not significant, n = 7). (F) HE staining of the three pairs of samples for single-cell RNA sequencing (scale bars = 50 μm). (G) tSNE plot showing the expression of genes related to tight junction in epithelial cells. (H) AB-PAS staining showing the mucus layer of the functional and defunctioned intestines (**P* < 0.05, n = 5, scale bars = 50 μm). (I) tSNE plots showing the expression of *SH2D6* and *DEFB1* in epithelial cells. (J) Violin plot showing the *DEFB1* expression in tuft cells of the functional and defunctioned intestines (ns = not significant, Wilcoxon rank-sum test). (K) Immunohistochemistry staining of PTGS1 showing tuft cells in the functional and defunctioned intestines (ns = not significant, n = 5, scale bars = 50 μm). E, H, K, paired Wilcoxon rank-sum test. All values are presented as mean ± SEM of each group. De, Defunctioned; Fu, Functional; AB-PAS, Alcian blue/periodic acid-schiff.

**FIGURE S3** Functional analysis of goblet cells and transcription factors analysis of epithelial cells. (A) and (B) Immunohistochemistry staining showing expression of MUC2 and TFF1 in functional and defunctioned intestine (**P* < 0.05, n =5, scale bars = 50 μm, paired Wilcoxon rank-sum test, all values are presented as mean ± SEM of each group). (C) and (D) Dot plot showing the expression of genes related to tight junction, antigen processing, mineral absorption and oxidative phosphorylation in goblet cells; violin plot showing different expression of genes related to oxidative phosphorylation in goblet cells of the functional and defunctioned intestines. (E) Dot plot showing the transcription factors that are significantly expressed in each subset of epithelial cells. (F) Volcano plots showing significant differential expression of transcription factors between goblet cells of the functional and defunctioned intestines (*P* < 0.05, Wilcoxon rank-sum test). (G) Gene set variation analysis showing the score of gene set (detection of mechanical stimulus) in each subset of epithelial cells. De, Defunctioned; Fu, Functional; EC, enterochromaffin.

**FIGURE S4** Crosstalk between fibroblasts and other cells. (A) Overview of ligand–receptor interactions between each cell type. Colors indicate the quantity of ligand-receptor pairs. (B) Detailed view of the ligand–receptor pairs expressed by fibroblasts and the other cell types. Numbers indicate the quantity of ligand–receptor pairs for each intercellular link. (C–E) Gene set enrichment analysis showing enriched pathways in macrophages and mast cells of the functional or defunctioned intestines. (F) and (G) Dot plot showing ligand–receptor interactions between fibroblasts and epithelial cells in the functional and defunctioned intestines. Color indicates permutation p-value and point size indicates the scaled mean expression level of ligand and receptor. De, Defunctioned; Fu, Functional.

**FIGURE S5** Joint analysis of single-cell data and Crohn's disease (CD)-related public databases. (A) Dot plot showing the log2 fold change in the expression of genes significantly altered in mast cells, glial cells, blood endothelial cells and lymphatic endothelial cells of the defunctioned intestine and in CD tissues (*P* < 0.05). Colors indicate the upregulation (red) or downregulation (blue) in gene expression in the defunctioned intestines. (B) tSNE plots showing the clustering results and monocyte signature scores in GSE134809. The clustering was performed using Seurat and the score was evaluated using “AddModuleScore” in Seurat with default parameter. Cluster5 has high monocyte signature scores. (C–D) UMAP plots showing the clustering results, groups and monocyte signature scores of cluster5. (E) UMAP plots showing the expression of marker genes of monocyte and inflammatory monocytes/macrophage in cluster5. (F) Violin plot showing the monocyte signature scores of subsets of cluster5. (G) Violin plot showing the monocyte signature scores in normal and CD tissues (*P* < 0.001, Wilcoxon rank-sum test). (H) Correlation between monocyte signature score and microbial taxa in CD datasets (HMP2) with paired tissue sequencing and 16S rRNA sequencing. Spearman's rank correlation was used for analysis (*P* < 0.05). De, Defunctioned; Fu, Functional; NC, Normal control.

**Table S1** Clinical characteristics of the patients

**Table S2** Quality filtering information

**Table S3** Clustering results of all cells

**Table S4** Markers of each subset of T, Myeloid-mast, stromal and epithelial cells

**Table S5** Clustering results of T cells

**Table S6** Clustering results of myeloid-mast cells

**Table S7** Clustering results of stromal cells

**Table S8** Clustering results of epithelial cells

**Table S9** Information of primer sequences

**Table S10** Crohn's disease (CD)-related genes
